# Identification of prognostic genes in uveal melanoma microenvironment

**DOI:** 10.1371/journal.pone.0242263

**Published:** 2020-11-16

**Authors:** Huan Luo, Chao Ma

**Affiliations:** 1 Charité–Universitätsmedizin Berlin, Corporate Member of Freie Universität Berlin, Humboldt-Universität zu Berlin, and the Berlin Institute of Health, Berlin, Germany; 2 Klinik für Augenheilkunde, Charité–Universitätsmedizin Berlin, Corporate Member of Freie Universität Berlin, Humboldt-Universität zu Berlin, and Berlin Institute of Health, Berlin, Germany; 3 BCRT—Berlin Institute of Health Center for Regenerative Therapies, Charité—Universitätsmedizin Berlin, Berlin, Germany; University of Queensland Diamantina Institute, AUSTRALIA

## Abstract

**Background:**

Uveal melanoma (UM) is the most common primary intraocular malignancy in adults. Many previous studies have demonstrated that the infiltrating of immune and stromal cells in the tumor microenvironment contributes significantly to prognosis.

**Methods:**

Dataset TCGA-UVM, download from TCGA portal, was taken as the training cohort, and GSE22138, obtained from GEO database, was set as the validation cohort. ESTIMATE algorithm was applied to find intersection differentially expressed genes (DEGs) among tumor microenvironment. Kaplan-Meier analysis and univariate Cox regression model were performed on intersection DEGs to initial screen for potential prognostic genes. Then these genes entered into the validation cohort for validation using the same methods as that in the training cohort. Moreover, we conducted correlation analyses between the genes obtained in the validation cohort and the status of chromosome 3, chromosome 8q, and tumor metastasis to get prognosis genes. At last, the immune infiltration analysis was performed between the prognostic genes and 6 main kinds of tumor-infiltrating immune cells (TICs) for understanding the role of the genes in the tumor microenvironment.

**Results:**

959 intersection DEGs were found in the UM microenvironment. Kaplan-Meier and Cox analysis was then performed in the training and validation cohorts on these DEGs, and 52 genes were identified with potential prognostic value. After comparing the 52 genes to chromosome 3, chromosome 8q, and metastasis, we obtained 21 genes as the prognostic genes. The immune infiltration analysis showed that Neutrophil had the potential prognostic ability, and almost every prognostic gene we had identified was correlated with abundances of Neutrophil and CD8+ T Cell.

**Conclusions:**

Identifying 21 prognosis genes (SERPINB9, EDNRB, RAPGEF3, HFE, RNF43, ZNF415, IL12RB2, MTUS1, NEDD9, ZNF667, AZGP1, WARS, GEM, RAB31, CALHM2, CA12, MYEOV, CELF2, SLCO5A1, ISM1, and PAPSS2) could accurately identify patients' prognosis and had close interactions with Neutrophil in the tumor environment, which may provide UM patients with personalized prognosis prediction and new treatment insights.

## Introduction

Uveal melanoma (UM) accounts for 3% -5% of all melanomas and is the most common primary intraocular malignancy in adults. UM comprises approximately 95 percent of melanomas from the eye, with the remainder arising from the conjunctiva [1]. UM usually appears asymptomatic and is found during routine eye examinations. About half of patients will develop visual symptoms such as flashing, floating objects, or visual field defects [2]. About 20% to 30% of patients diagnosed with primary UM die from systemic metastases within five years of diagnosis, and 45% die within 15 years of initial diagnosis [3]. American Cancer Society reported that when UM was spread to distant parts of the body, the 5-year relative survival rate was about 13% [4]. The median age at diagnosis is about 62 years, but the peak of the diagnosis ranges from 70 to 79 years [5]. No therapy has been shown to improve overall survival for patients with UM [5]. Further understanding of the molecular pathogenesis of UM can provide vital information for exploring prognostic factors [5].

The tumor microenvironment is the environment surrounding the tumor, including surrounding blood vessels, immune cells, fibroblasts, signaling molecules, and extracellular matrix [6]. The tumor is closely related to the surrounding microenvironment and continuously interacts and together promotes the tumor's immune escape, growth, and metastasis, which all reflect the evolutionary nature of the tumor [6, 7]. It is reported that the level of immune cell infiltration is related to the prognosis. The activity of immune cells and stromal cells has been shown to predict the overall survival of cancer [8]. The inflammatory phenotype of UM is characterized by high infiltration of lymphocytes and macrophages and by the expression of human leukocyte antigen (HLA) Class I and II antigens [9]. Narasimhaiah and colleagues found that UM with IFNγ‐signature had a poorer prognosis and showed increased infiltration of CD8+ T lymphocytes and macrophages. In UM, it was demonstrated that immune cell infiltration was associated with poorer outcomes in the intermediate-risk group and increased in high‐risk group (73.7%) [10]. ESTIMATE, designed by Yoshihara et al., is a tool for predicting tumor purity, and the presence of infiltrating stromal/immune cells in tumor tissues using gene expression data [11]. Based on the ESTIMATE algorithm, the researchers obtained more possibilities to evaluate and explore the genetic changes of malignant tumors [12–14]. However, the distribution of immune and stromal scores in UM, and whether the ESTIMATE algorithm can be used to investigate the prognosis of patients with UM remains elucidated.

During the past few decades, genetic or epigenetic alterations have been confirmed to be associated with the tumorigenesis and progression of UM [15]. Gene mutations and chromosomal copy number variations are closely related to UM prognosis. According to reports, GNAQ and GNA11 mutations can promote cell proliferation and metastasis [16]. The loss of one copy of chromosome 3 (monosomy 3) in UM is associated with an increased risk of metastasis and poor prognosis [17]. Other chromosomal abnormalities have been shown to correlate with poor prognosis, including 8q gain, 6q loss, lack of 6p gain, 1p loss, and 16q loss [17–21]. Therefore, further exploration of gene mutation and copy number variation in UM can provide incisive information for prognosis.

To better understand the molecular pathogenesis of UM, in the present study, we used the ESTIMATE algorithm in conjunction with TCGA and GEO databases, along with the comparison with the status of chromosomal copy number variations to discover potential markers in the UM microenvironment.

## Materials and methods

### Data mining from TCGA and GEO

The gene expression profiles of UM from 80 patients and their clinical and survival data were downloaded from TCGA Xena Hub (https://tcga.xenahubs.net) with cohort name: TCGA Ocular melanomas (TCGA-UVM). Also, we researched the GEO database (https://www.ncbi.nlm.nih.gov/geo/) by setting a filter: 1) more than 60 cases; 2) with expression profiling data; 3) with survival data. At last, we selected dataset GSE22138, which contains 63 UM cases, for the study. In our research, TCGA-UVM was used as training cohort, while GSE22138 was taken as validation cohort.

### Immune and stromal scores

Immune scores and stromal scores of each case of the training cohort were calculated by the ESTIMATE algorithm R package named "ESTIMATE" (https://bioinformatics.mdanderson.org/public-software/estimate/, **S1 Table**) [11].

### Identification of the intersection differentially expressed genes (DEGs) among immune and stromal scores

According to their scores based on the median, all training cohort cases were divided into groups of high and low scores. DEGs were identified between high and low immune/stromal score groups using "limma" R package [22], with a cutoff of |log2(fold-change) | > 1 and false discovery rate (FDR) < 0.05. "pheatmap" R package was applied to produce heatmaps and clustering of DEGs. Genes that were up-regulated in both high immune and stromal scores groups were defined as intersection-up-regulated DEGs. Genes that were down-regulated in both high immune and stromal scores groups were taken as intersection-down-regulated DEGs. A combination of these two intersection DEGs was the intersection DEGs. Besides, the Metascape web tool (https://metascape.org/) was applied to perform Gene Ontology (GO) and Kyoto Encyclopedia of Genes and Genomes (KEGG) enrichment analysis on the intersection DEGs [23].

### Identification and validation of the potential prognostic genes

In the training cohort, Kaplan-Meier analysis was used to screen for potential prognostic genes from the intersection DEGs identified in the previous step based on overall survival. Only genes with p-value < 0.01 in the log-rank test were considered significant to pass Kaplan-Meier analysis screening. Also, univariate Cox regression analysis was performed on the training cohort to look for prognostic genes from the intersection DEGs with p-value < 0.01. Same as before, only genes that showed significant in the overall survival analysis were considered to pass univariate Cox regression analysis screening. The genes passed both Kaplan-Meier and univariate Cox analyses in the training cohort were then entered into the validation cohort for validation. The same methods were conducted like that in the training cohort. Only genes both passed the Kaplan–Meier and univariate Cox analyses with the cutoff p-value < 0.001 were able to move to the next step.

### Screen prognostic genes based on correlation with chromosome 3, chromosome 8q, and metastasis

In UM, chromosomal aberrations and gene mutations are closely related to treatment options and prognosis [17]. Moreover, metastasis is a strong predictor of the adverse outcome. Only a fraction of patients with UM metastasis survive, and almost all metastases die [24]. In Robertson's research, the status of chromosome 3 and 8q of each patient in the TCGA-UVM cohort has been studied and specifically described [17]. The Spearman rank correlation coefficient was applied to assess the correlations between the expression of each potential prognostic gene identified in the previous step and the copy number aberrations, as well as the metastasis status. Only genes passed all the correlation tests were taken as prognostic genes. P-value < 0.05 was considered statistically significant.

### Correlation of prognostic genes with the abundances of six kinds of tumor-infiltrating immune cells (TICs)

TIMER web server [25, 26] (https://cistrome.shinyapps.io/timer/) is a comprehensive resource for systematical analysis of immune infiltrates across diverse cancer types. The abundances of six immune infiltrates (B cells, CD4+ T cells, CD8+ T cells, Neutrophils, Macrophages, and Dendritic cells) are estimated by TIMER algorithm. The TIMER web server was applied to estimate the correlations between the abundances of TICs and the prognosis of UM via methods of Kaplan-Meier, univariate Cox, and multivariate Cox analysis. The correlations between abundance of each TIC and each prognostic gene were calculated in TIMER and were visualized via the "canvasXpress" R package. P-value < 0.05 was considered statistically significant.

## Results

### Clinical characteristics

**Fig 1:**
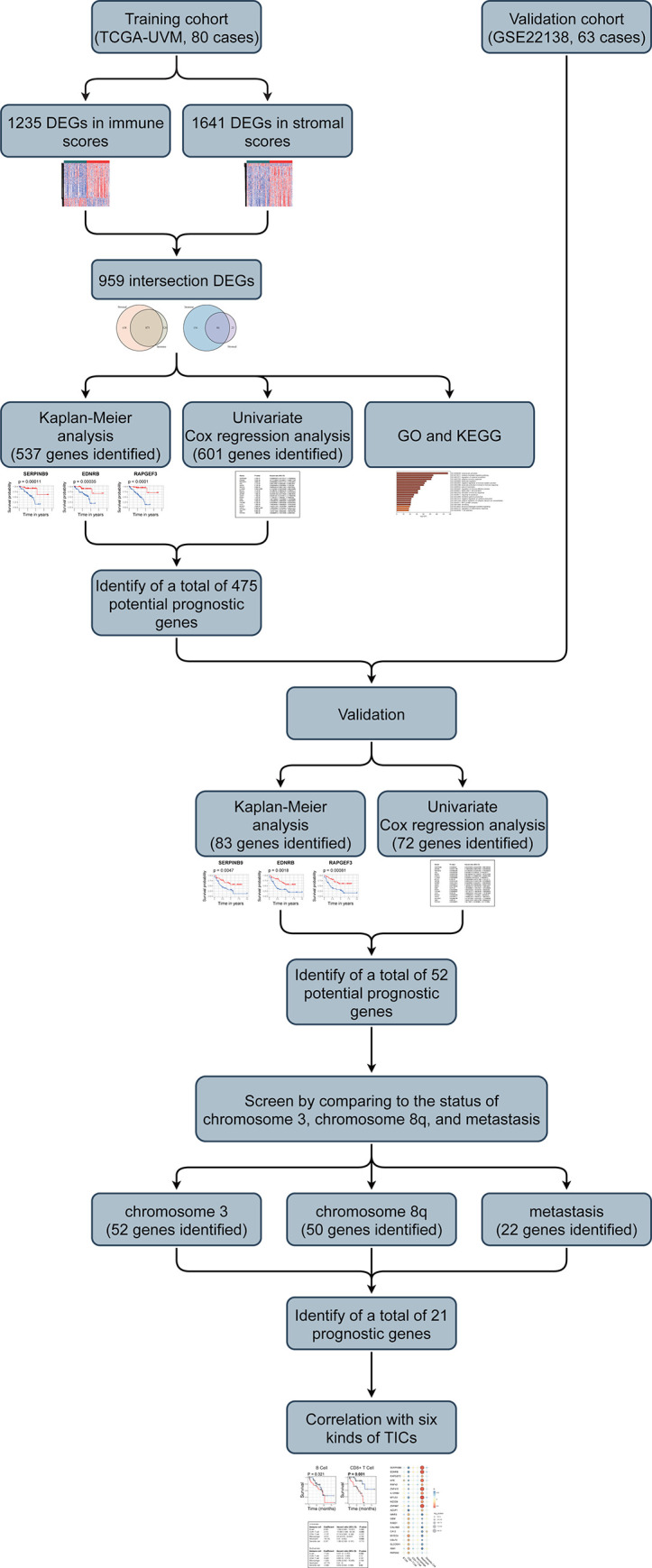
Flow chart of the study. The study was carried out in TCGA-LUAD and GSE72094 cohorts. The potential prognosis genes were obtained from training cohort and the validation cohort. Then the correlation analysis between the potential genes and the status of chromosome 3 and 8q and tumor metastasis were performed for prognosis genes. At last, the immune infiltration analysis was conducted. DEGs: differentially expressed genes; GO: Gene Ontology; KEGG: Kyoto Encyclopedia of Genes and Genomes; TICs: tumor-infiltrating immune cells.

The present research's flowchart is shown in **Fig 1**. 80 UM cases from TCGA-UVM were taken as the training cohort. The dataset GSE22138 with 63 UM patients was used as the validation cohort. The detailed clinical characteristics of both cohorts were summarized in **Table 1**.

**Table 1:** Clinical characteristics of patients involved in the study.

Characteristics		Training cohort (TCGA-UVM, n = 80)	Validation cohort (GSE22138, n = 63)
Age at diagnosis, years			
	<60	36 (45.00%)	28 (44.44%)
	≥60	44 (55.00%)	35 (55.56%)
	unknown	0 (0.00%)	0 (0.00%)
Gender			
	Female	35 (43.75%)	24 (38.10%)
	Male	45 (56.25%)	39 (61.90%)
	unknown	0 (0.00%)	0 (0.00%)
Stage			
	I	0 (0.00%)	NA
	II	36 (45.00%)	NA
	III	40 (50.00%)	NA
	IV	4 (5.00%)	NA
	unknown	0 (0.00%)	NA
T classification			
	T1	0 (0.00%)	NA
	T2	4 (5.00%)	NA
	T3	36 (45.00%)	NA
	T4	38 (47.50%)	NA
	unknown	2 (2.50%)	NA
N classification			
	N0	76 (95.00%)	NA
	N1	0 (0.00%)	NA
	unknown	4 (5.00%)	NA
M classification			
	M0	51 (91.25%)	28 (44.44%)
	M1	4 (3.75%)	35 (55.56%)
	unknown	25 (5.00%)	0 (0.00%)

### Intersection DEGs based on immune and stromal scores

**Fig 2:**
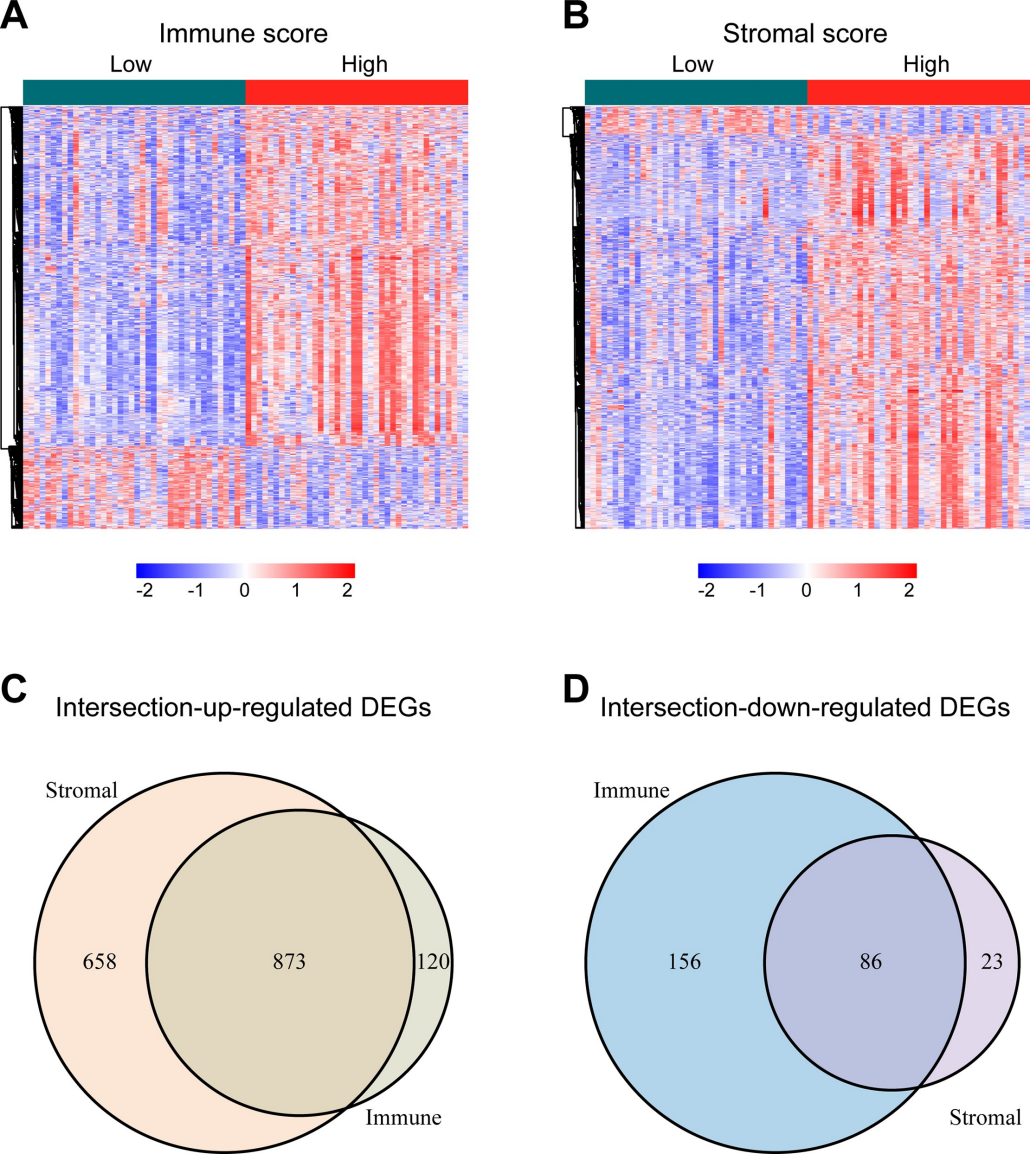
Identification of the intersection DEGs among immune and stromal scores in UM. **(A)** Heatmap of the DEGs of immune scores of top half (high score) vs. bottom half (low score). (Cutoff: |log2(fold-change) |> 1, FDR < 0.05). **(B)** Heatmap of the DEGs of stromal scores of top half (high score) vs. bottom half (low score). (Cutoff: |log2(fold-change) |> 1, FDR < 0.05). **(C, D)** Venn diagrams showing the number of intersection-up-regulated DEGs (**C**) or intersection-down-regulated DEGs (**D**) in stromal and immune score groups. Heatmaps were drawn based on the average method and correlation distance measurement method. Genes with higher expression are shown in red, lower expression are shown in blue, genes with same expression level are in white. DEGs: differentially expressed genes; UM: uveal melanoma; FDR: false discovery rate.

**Fig 3:**
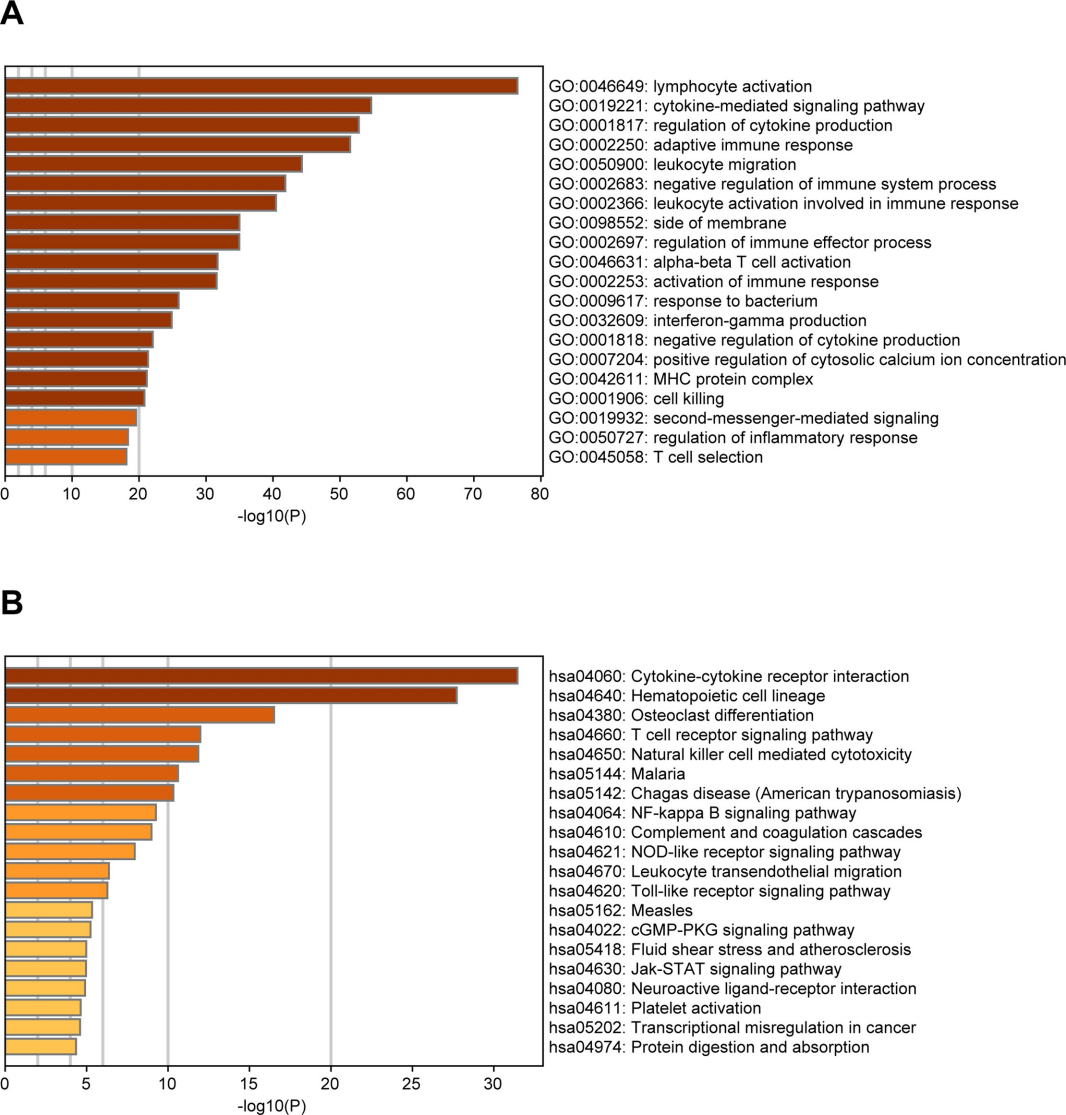
Bar graph of enriched GO **(A)** and KEGG **(B)** terms across the intersection DEGs in UM, colored by p-values. GO: Gene Ontology; KEGG: Kyoto Encyclopedia of Genes and Genomes; DEGs: differentially expressed genes; UM: uveal melanoma.

For identifying the DEGs among immune and stromal scores, cases in the training cohort were divided into groups of high and low scores according to their scores based on the median, and the DEG analysis was performed using the "limma" R package. **Fig 2A** shows a heatmap of 1235 DEGs between immune score groups. **Fig 2B** displays a heatmap consisting of 1641 DEGs between stromal score groups. Via integrated bioinformatics analysis, we identified 873 intersection-up-regulated DEGs (**Fig 2C**) and 86 intersection-down-regulated DEGs (**Fig 2D**). Our subsequent research focused on these 959 intersection DEGs (**S2 Table**). As shown in **Fig 3A**, the enriched GO terms in the intersection DEGs were mainly related to lymphocyte activation, cytokine-mediated signaling pathway, cytokine production regulation, adaptive immune response, and leukocyte migration. And the KEGG terms were mostly focused on Cytokine-cytokine receptor interaction, Hematopoietic cell lineage, Osteoclast differentiation, T cell receptor signaling pathway, and Natural killer cell mediated cytotoxicity (**Fig 3B**).

### Identification and validation of the potential prognostic genes

Kaplan-Meier and univariate Cox regression analysis were performed on 80 UM patients in the training cohort to assess the prognostic relationship between the 959 intersection DEGs and overall survival. 537 genes were extracted from the Kaplan-Meier analysis, while 601 genes were identified as significant in the Cox regression analysis. Taking together, 475 genes in the intersection of the two results are defined as genes with prognostic value for subsequent analysis (**S3 Table**). Also, we put the 475 genes into the validation cohort for validation using the same methods as that in the training cohort. 83 genes were found prognosis value via Kaplan-Meier analysis, and 72 genes were seen holding capacity of predicting the outcome by Cox regression analysis. Finally, 52 potential prognosis genes were discovered (**S4 Table**).

### Screening prognostic genes from comparing the status of chromosome 3, chromosome 8q, and metastasis to the potential prognostic genes

**Fig 4:**
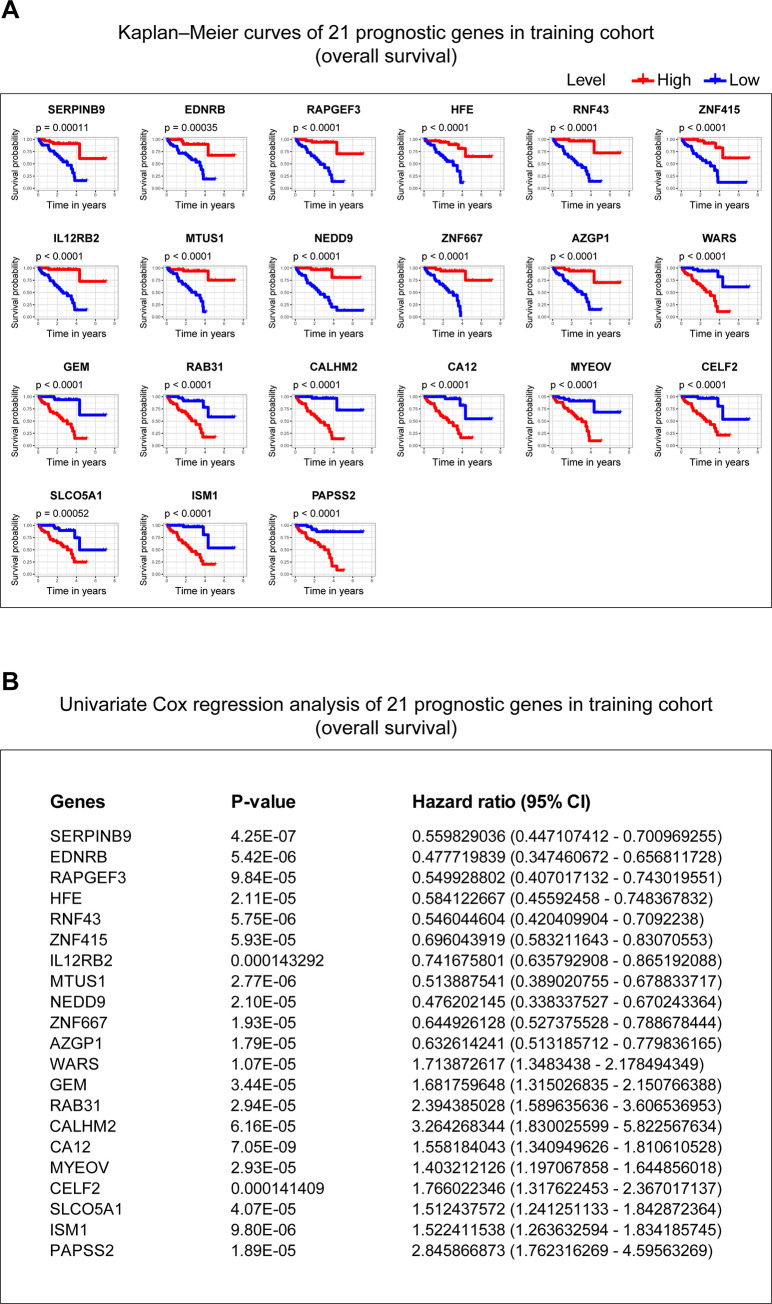
Kaplan–Meier curves and univariate Cox analysis of 21 genes in the training cohort. **(A)** The Kaplan–Meier curves of 21 genes in the training cohort. P-value was examined in the Log rank test. **(B)** The univariate Cox analysis of 21 genes in the training cohort. P-value < 0.01 is considered statistically significant. 95% CI: 95% Confidence Interval.

**Fig 5:**
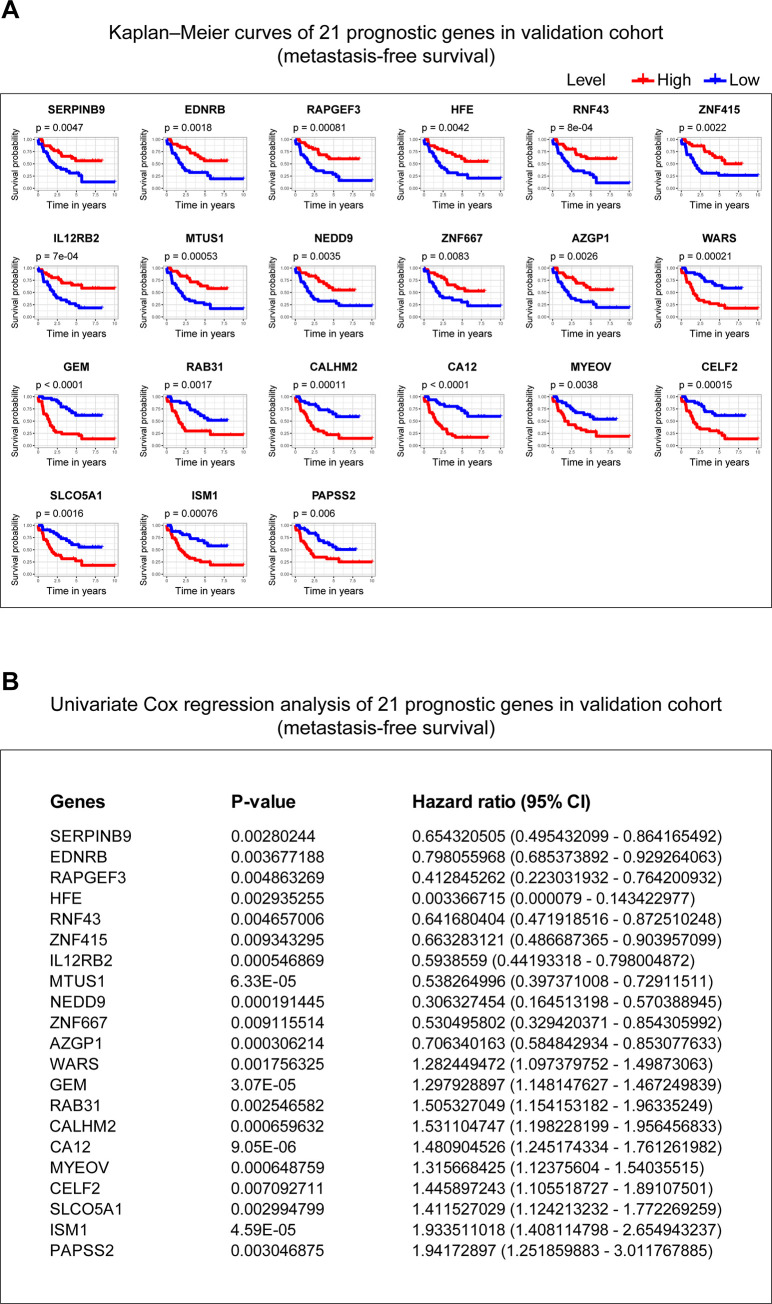
Kaplan–Meier curves and univariate Cox analysis of 21 genes in the validation cohort. **(A)** The Kaplan–Meier curves of 21 genes in the validation cohort. P-value was examined in the Log rank test. **(B)** The univariate Cox analysis of 21 genes in the validation cohort. P-value < 0.01 is considered statistically significant. 95% CI: 95% Confidence Interval.

Furthermore, to find prognostic genes in UM, we performed correlation analyses to assess the relationship between the 52 potential prognosis genes and the status of chromosome 3, chromosome 8q, and metastasis in TCGA-UVM cohort. 5 genes locate in chromosome 3 or 8, including ALDH1L1, locating in chromosome 3; GEM, MTUS1, RIMS2, SLCO5A1, locating in chromosome 8 (**S4 Table**). Spearman test was used to assess the correlation between copy chromosome numbers, metastasis and the potential prognosis genes. The results showed that 52 genes were significantly correlated with copy numbers of chromosome 3, 50 genes were significantly correlated with copy numbers of chromosome 8q, and 22 genes were significantly correlated with tumor metastasis. Combining the above three results, 21 genes in the intersection (0 genes locate in chromosome 3, while 3 genes, including GEM, MTUS1, and SLCO5A1 locate in chromosome 8), were identified as prognostic genes (**Table 2**). The Kaplan–Meier curves and univariate Cox analysis of 21 genes in the training cohort (**Fig 4**) and the validation cohort (**Fig 5**) were plotted.

**Table 2:** 21 prognostic genes identified in this study.

Gene	chromosome 3		chromosome 8q		metastasis		Genomic location (GRCh38/hg38)
	R	P-value	R	P-value	R	P-value	
SERPINB9	0.641	1.49E-10	-0.334	2.46E-03	-0.362	6.67E-03	chr6:2,887,265–2,903,312
EDNRB	0.790	3.00E-18	-0.467	1.25E-05	-0.326	1.50E-02	chr13:77,895,481–77,975,529
RAPGEF3	0.613	1.48E-09	-0.520	7.71E-07	-0.313	1.99E-02	chr12:47,734,363–47,771,040
HFE	0.668	1.32E-11	-0.656	3.90E-11	-0.304	2.39E-02	chr6:26,087,281–26,098,343
RNF43	0.697	6.66E-13	-0.444	3.69E-05	-0.300	2.61E-02	chr17:58,352,500–58,417,620
ZNF415	0.619	9.47E-10	-0.513	1.17E-06	-0.296	2.85E-02	chr19:53,107,879–53,133,077
IL12RB2	0.633	2.93E-10	-0.437	5.13E-05	-0.287	3.38E-02	chr1:67,307,351–67,397,090
MTUS1	0.682	3.17E-12	-0.652	5.79E-11	-0.287	3.38E-02	chr8:17,643,794–17,801,220
NEDD9	0.703	3.68E-13	-0.350	1.45E-03	-0.278	4.00E-02	chr6:11,183,298–11,382,348
ZNF667	0.628	4.33E-10	-0.521	7.06E-07	-0.278	4.00E-02	chr19:56,438,512–56,478,065
AZGP1	0.661	2.47E-11	-0.526	5.30E-07	-0.269	4.70E-02	chr7:99,966,720–99,976,112
WARS	-0.677	5.25E-12	0.562	5.81E-08	0.269	4.70E-02	chr14:100,333,788–100,376,805
GEM	-0.736	7.18E-15	0.595	5.92E-09	0.278	4.00E-02	chr8:94,249,253–94,262,350
RAB31	-0.705	2.80E-13	0.572	3.02E-08	0.282	3.68E-02	chr18:9,708,231–9,862,556
CALHM2	-0.764	1.65E-16	0.580	1.78E-08	0.296	2.85E-02	chr10:103,446,785–103,452,405
CA12	-0.559	6.90E-08	0.467	1.25E-05	0.300	2.61E-02	chr15:63,321,378–63,382,110
MYEOV	-0.720	5.29E-14	0.577	2.15E-08	0.300	2.61E-02	chr11:69,294,138–69,367,726
CELF2	-0.709	1.96E-13	0.446	3.33E-05	0.318	1.82E-02	chr10:10,798,397–11,336,675
SLCO5A1	-0.738	5.57E-15	0.517	9.13E-07	0.331	1.36E-02	chr8:69,667,046–69,835,064
ISM1	-0.742	3.40E-15	0.581	1.55E-08	0.340	1.12E-02	chr20:13,221,274–13,300,651
PAPSS2	-0.618	9.99E-10	0.551	1.16E-07	0.406	2.12E-03	chr10:87,659,613–87,747,705

### Correlation of prognostic genes identified with the abundances of six kinds of TICs

**Fig 6:**
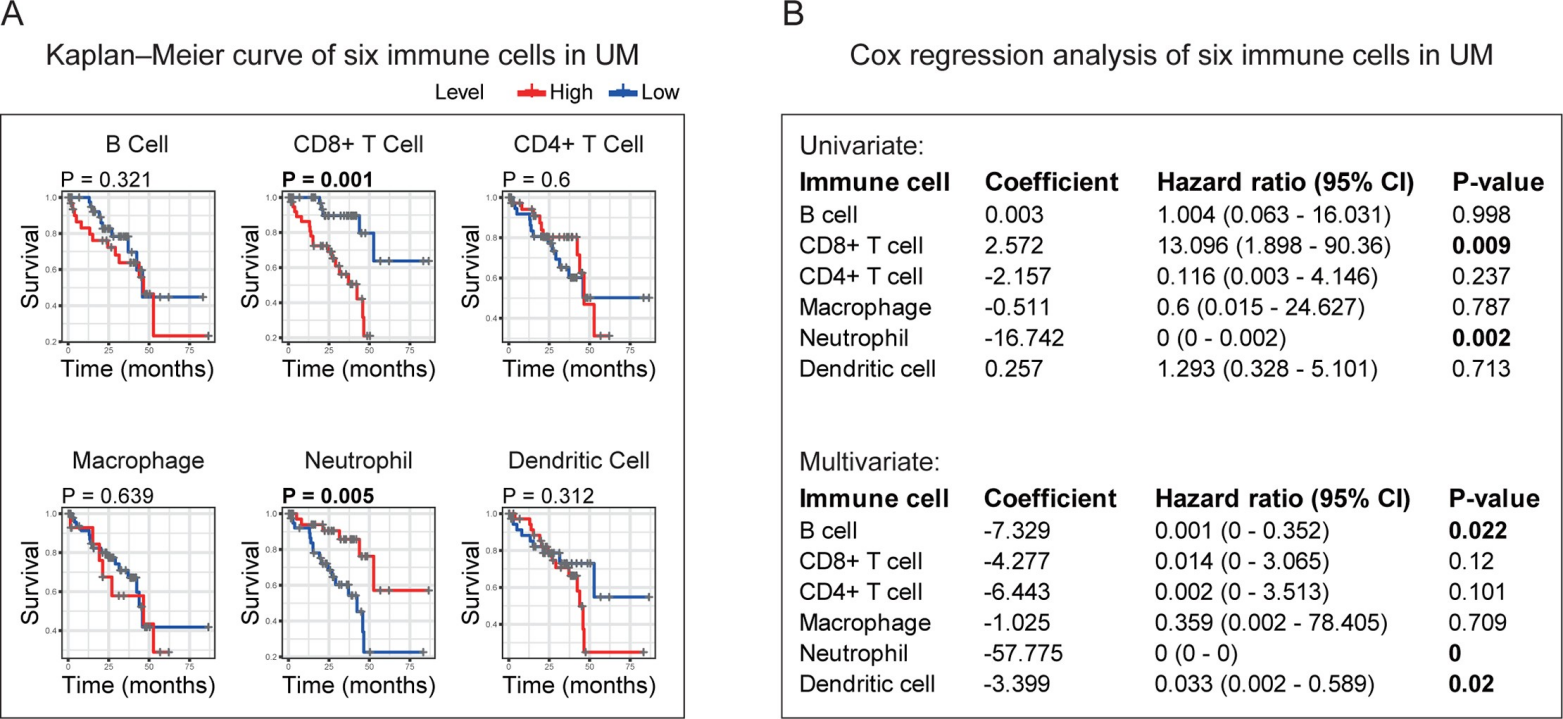
Identification of the prognostic value of each TIC based on the 6 TICs infiltration volume and survival data. **(A)** Kaplan–Meier survival curves of 6 TICs in UM. **(B)** Univariate and multivariate Cox analysis based on each of the 6 TICs infiltration volume and overall survival. All p-values were calculated using Cox regression hazards analysis. The p-value in bold represents statistical significance (p-value < 0.05). TICs: tumor-infiltrating immune cells; UM: uveal melanoma.

First, we examined the impacts of six immune cells on the prognosis of UM. Kaplan–Meier, univariate Cox, and multivariate Cox methods were applied to determine whether each type of immune cell can influence the UM prognosis. Kaplan–Meier curves indicated that CD8+ T cell and Neutrophil hold the capacity to predict UM outcome (**Fig 6A**). In univariate Cox analysis results, CD8+ T cell and Neutrophil were found to have predictive ability (**Fig 6B**). As shown from the multivariate Cox test, B cell, Neutrophil, and Dendritic cell owned the prognostic power (**Fig 6B**). Based on the above results, we could see that only Neutrophil maintained a significant predictive value in all three tests. Neutrophil was acting as a potential prognostic immune cell in UM microenvironment.

**Fig 7:**
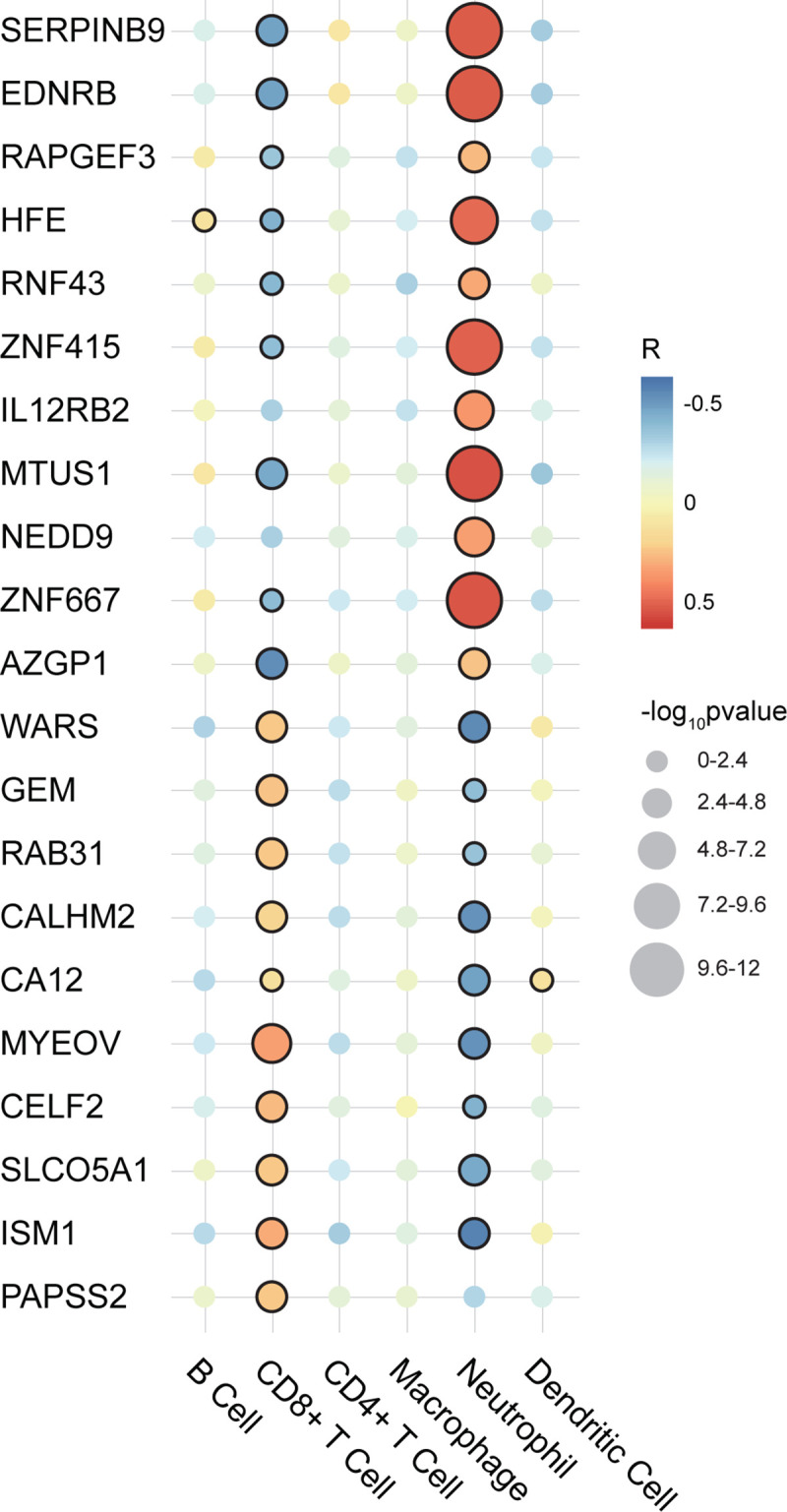
Correlation analysis between 21 prognostic genes and 6 TICs in UM. The illustration is on the right. The larger the circle, the smaller the p-value and the more significant the correlation. A circle with a black edge represents statistical significance (p-value < 0.05). R: correlation coefficient; TICs: tumor-infiltrating immune cells; UM: uveal melanoma.

Next, we checked the correlation between each prognostic gene and each TIC in UM. As shown in **Fig 7**, almost all prognostic genes (except PAPSS2) were related to Neutrophil infiltration. Among them, SERPINB9, EDNRB, RAPGEF3, HFE, RNF43, ZNF415, IL12RB2, MTUS1, NEDD9, ZNF667, and AZGP1 were positively correlated with Neutrophil infiltration, while WARS, GEM, RAB31, CALHM2, CA12, MYEOV, CELF2, SLCO5A1, and ISM1 were negatively correlated with Neutrophil infiltration. Besides, we found significant correlations also occurred in the relationship between prognostic genes (except IL12RB2 and NEDD9) and CD8+ T Cell. Interestingly, the correlation here with CD8+ T Cell infiltration is opposite to that in Neutrophil. As shown in **Fig 7**, SERPINB9, EDNRB, RAPGEF3, HFE, RNF43, ZNF415, MTUS1, ZNF667, and AZGP1 were negatively correlated with CD8+ T Cell, and WARS, GEM, RAB31, CALHM2, CA12, MYEOV, CELF2, SLCO5A1, ISM1, and PAPSS2 were positively correlated with CD8+ T Cell infiltration. In addition to the correlations found above, other correlations only existed between HFE and B Cell, and between CA12 and Dendritic Cell.

Overall, the analysis results showed that Neutrophil had the potential prognostic ability, and almost every prognostic gene we had identified was correlated with the infiltration of Neutrophil and CD8+ T Cell.

## Discussion

In the present study, we identified 21 UM prognostic genes from tumor microenvironment by comprehensively analyzing the TCGA and GEO. By discovering the DEGs among tumor microenvironment and investigating the potential prognosis of DEGs using Kaplan-Meier and univariate Cox analyses in the training cohort, we obtained 475 genes that were pronounced related to outcome. By applying these genes in the validation cohort screened by Kaplan-Meier and univariate Cox analyses, 52 genes were validated holding potential prognostic value. What is more important, we compared these 52 genes to the status of chromosome 3, chromosome 8q, and tumor metastasis, to get prognostic genes. Finally, 21 genes were identified as prognosis genes in our study. To clarify the relationship between these prognostic genes and immune infiltration, we conducted an immune infiltration analysis with the 21 genes and 6 main TICs, finding that the correlation between Neutrophil and 21 genes potentially acted as one of the factors that contribute to the prognosis capacity of the 21 genes. On the “road” to find the prognostic genes of UM, we are the first to combine tumor microenvironment scores and double screening (Kaplan-Meier and univariate Cox methods) for training and introduce chromosome copy number variation for gene screen. Such work we have done aimed to guide future research in UM.

Cancer is not only a cluster of malignant cells but also a complex "rogue" organ. Many other cells are recruited into these organs and may be destroyed by transformed cells. The interaction between malignant and non-transformed cells creates the tumor microenvironment [27]. The presence of immune cells infiltrating in and around tumors and their relationship with clinical outcomes have led to the hypothesis that the immune microenvironment is an important prognostic factor for cancer [28, 29]. Tumor-infiltrating immune cells have been reported to correlate with clinical prognosis in various tumors like hepatocellular carcinoma [30], colorectal cancer [31], gastric cancer [32]. The eye is an immune-privileged site, but inflammation can exist in the established ocular tumor microenvironment [33, 34]. One recent study has demonstrated that loss of BAP1 expression is strongly associated with immune modulation of the microenvironment, and it makes an impact on the immunotherapy of UM [35]. Furthermore, Narasimhaiah et al. found that immune cell infiltration was associated with poorer outcomes in the intermediate-risk group and increased in the high‐risk group, indicating that immune infiltration may be a promising biomarker repository for better-personalized management of UM [10]. Our research on genes involved in the microenvironment of UM provides an opportunity for the development of therapeutic agents or gene targets.

Studies showed that chromosome aberrations and gene mutations in UM are closely related to clinical results. The loss of a chromosome 3 in UM is associated with an increased risk of metastasis and poor prognosis [17, 20]. Previous studies have shown that besides chromosome 3, the increase in chromosome 8q is also related to poor survival prognosis [36–39]. Moreover, metastasis is a strong predictor of the bad outcome. Only a fraction of patients with UM metastasis survive [24]. Another report demonstrated that up to 50% of patients diagnosed with uveal melanoma would die of metastasis after treatment of the tumor [24]. To make our research more robust, we performed the Spearman test to assess the correlation between the above-mentioned influential factors and the expression level of potential prognosis genes obtained from the validation cohort to further screen prognostic genes in this study (**Table 2**). In addition to chromosome 3 and 8q, other chromosomal abnormalities have been shown to correlate with poor prognosis and these include 6q loss, lack of 6p gain, 1p loss, and 16q loss [17–21]. Among the 21 prognostic genes found in this study, 7 genes (SERPINB9, HFE, IL12RB2, MTUS1, NEDD9, GEM, and SLCO5A1) were located in the chromosomes as mentioned above (**Table 2**). These 7 genes potentially affect the chromosome variation, leading to the occurrence and development of UM, but how they affect the UM is still unknown and needs to be ascertained.

Specifically, our study identified 21 prognostic genes in UM. SERPINB9, EDNRB, RAPGEF3, HFE, RNF43, ZNF415, IL12RB2, MTUS1, NEDD9, ZNF667, and AZGP1 indicated a favorable prognosis, while, WARS, GEM, RAB31, CALHM2, CA12, MYEOV, CELF2, SLCO5A1, ISM1, and PAPSS2 suggested a poor outcome. EDNRB is a 7-span transmembrane G-protein coupled receptor, and since membrane-located receptors constitute approximately 45% of all therapeutic drug targets [40]. A study showed that EDNRB expression is reduced in large primary UM and small cell lung cancer with high metastatic genotype and phenotype. The decreased expression of EDNRB in large primary UM is related to early clinical metastasis and short survival [41]. WARS is a member of the aminoacyl-tRNA synthase family [42], also known as TRPRS, WRS, which is a potential prognostic marker of metastasis [43]. WARS has been found to be unbalanced in a variety of cancers (such as oral cancer, ovarian cancer, pancreatic cancer, colorectal cancer, etc.) [44–49]. A recent study showed that the expression of WARS is up-regulated in UM cells and is related to the poor prognosis of UM patients. WARS may partially promote the growth of UM cells by activating the PI3K/AKT signaling pathway, thereby accelerating tumor development [44]. In addition to EDNRB and WARS, the remaining 19 genes showing evidence interacting in the progress of various types of cancer [50–68], but left few traces showing the connection with UM prognosis in the previous research. Besides our findings, LAG3 was defined as a potential candidate for immune checkpoint blockade in patients with high risk UM in a recent study [69]. LAG3 is expressed on natural killer cells, B cells, and dendritic cells. In addition, it is also expressed on the cell membrane of tumor-infiltrating lymphocytes, activated CD4 + and CD8 + T cells, and regulatory T cells [70]. LAG3 may be a very promising immune checkpoint. It is a co-inhibitory receptor that suppresses T cell activation and cytokine secretion, thus ensuring immune homeostasis [71]. Targeted LAG3 immunotherapy is moving forward in active clinical trials, and the combined immunotherapy of anti-LAG3 and anti-PD-1 has shown exciting effects in combating PD-1 resistance [70].

The immune infiltration analysis showed that Neutrophil infiltration had the most potential prognostic capacity among 6 kinds of TICs in UM, and almost all the 21 genes identified were correlated with Neutrophil infiltration. This finding indicated that the significant correlation between Neutrophil and 21 genes potentially acted as one of the factors that contribute to the prognosis capacity of the 21 genes. The analysis also found almost every prognostic gene correlating with CD8+ T cell. However, CD8+ T cell did not show prognostic ability in UM based on our analysis. The tumor-promoting effects of neutrophils are mediated by different mechanisms. Neutrophils play an important role in angiogenesis by expression of matrix metallo-proteases, such as MMP9 [72, 73]. Besides, neutrophils inhibit the anti-tumor CD8 + T cell response by degranulation of granular constituents, production of ROS and release of arginase, and expression of PD-L1 [74]. It has recently been shown that in a mouse model of breast cancer, neutrophils inhibit the anti-tumor T cell response and play a key role in tumor metastasis [75]. However, how CD8 + T cells interplay with 21 genes and whether or how their relationship affects the prognosis of UM remains to be determined.

DecisionDx-UM is a prognostic test that determines the metastatic risk associated with UM [76]. Specifically, the assay determines the activity or "expression" of 15 genes, which indicate a patient's individual risk, or class. According to the report of the Collaborative Eye Oncology Group (COOG), the DecisionDx-UM GEP test is an accurate prospectively validated molecular classifier whose results are highly correlated with metastatic potential [77, 78]. In a prospective multicenter study, Plasseraud and colleagues demonstrated that the DecisionDx-UM could accurately predict the risk of metastasis in patients with UM [79].

Our research also has some limitations. This study is to find immune related biomarkers, which could give insight into immune modulation and potential clinical targets. However, compared with the seminal work of DecisionDx-UM performance on UM prognosis predicting, the presented work may remain limit. Although TCGA-UVM is a cohort that is currently recognized by most scholars, the data in it are from large uveal melanoma treated with enucleation. Similarly, the GSE22138 cohort, which was published online on the GEO database platform, and its academic recognition is also undoubted. Still, most of the data in it came from large eye tumors. Such sample distribution in these two cohorts may not be consistent with the clinical population. Therefore, our research may have a selection bias for database selection. It is unclear how many of those markers would actually hold up in a truly prospective study not relying on sequencing data from very large tumors. Our 21 prognosis genes came from retrospective data, and more prospective data were needed to prove its clinical utility. In addition, there is currently no wet experimental data explaining the relationship between these 21 genes and their mechanism in UM samples. Therefore, between the 21 genes and the prognosis of UM, more effort is needed to clarify the potential relationship.

## Conclusion

In conclusion, our research defined 21 prognosis genes in UM. It is a comprehensive analysis of the TCGA and the GEO database. These genes were related to the prognosis of UM and can accurately identify the outcome of patients. Notably, we adopted the comparison with the status of chromosomes 3 and 8q, and tumor metastasis to further screen prognosis genes. The immune infiltrating analysis revealed that the 21 genes had close interactions with Neutrophil, which may advance new therapies for UM treatment.

## Supporting information

S1 Table Immune and stromal scores of each sample of TCGA-UVM cohort. (PDF)

S2 Table 959 intersection differentially expressed genes between high and low scores. (PDF)

S3 Table 475 genes were significantly predicting the prognosis of UM patients in the training cohort by both Kaplan-Meier and univariate Cox regression analyses (p-value < 0.01). (PDF)

S4 Table 52 genes were significantly predicting the prognosis of UM patients in the validation cohort by both Kaplan-Meier and univariate Cox regression analyses (p-value < 0.01). (PDF)
