# Supplementary material for: Identification of prognostic genes in uveal melanoma microenvironment
Source: PLoS One. 2020 Nov 16;15(11):e0242263. doi: 10.1371/journal.pone.0242263 (PMC7668584; doi:10.1371/journal.pone.0242263)
Supplement: S1 Table — (PDF) [file pone.0242263.s001.pdf]

S1 Table. Immune and stromal scores of each sample of TCGA-UVM cohort.

ID	StromalScore	ImmuneScore	ESTIMATEScore
TCGA-VD-A8KM-01	-185.4095519	1704.279428	1518.869876
TCGA-V4-A9F4-01	-1029.591707	-1042.336117	-2071.927823
TCGA-WC-A881-01	-1283.217101	-921.5091353	-2204.726236
TCGA-V4-A9F1-01	-932.2540914	-396.3398323	-1328.593924
TCGA-VD-AA8N-01	-745.594673	116.2847118	-629.3099612
TCGA-V4-A9EY-01	-867.4359065	-525.5062604	-1392.942167
TCGA-V4-A9EI-01	-1378.805844	-793.2370604	-2172.042904
TCGA-VD-AA8O-01	-1321.909027	-8.777790588	-1330.686817
TCGA-WC-A880-01	-1390.010288	-999.585495	-2389.595783
TCGA-WC-A882-01	-565.1581264	-289.3840316	-854.5421581
TCGA-VD-AA8P-01	-1089.194136	-1098.429914	-2187.62405
TCGA-WC-AA9E-01	-1438.309537	-1131.440002	-2569.749539
TCGA-V4-A9F8-01	-924.9451692	-545.2106049	-1470.155774
TCGA-VD-A8K9-01	-1678.721217	-1236.363867	-2915.085085
TCGA-V4-A9EZ-01	-1297.485924	-580.4631479	-1877.949072
TCGA-VD-AA8Q-01	-876.253348	-192.5257646	-1068.779113
TCGA-V4-A9E9-01	-1566.266678	-1123.924451	-2690.191129
TCGA-VD-A8KL-01	-1250.547388	-1296.907568	-2547.454956
TCGA-WC-A87T-01	-1730.288441	-1467.068428	-3197.35687
TCGA-YZ-A980-01	-970.7241604	-1138.558073	-2109.282233
TCGA-VD-AA8M-01	-1255.351448	-884.836451	-2140.187899
TCGA-VD-AA8T-01	-1254.979012	-1043.34181	-2298.320821
TCGA-VD-A8KI-01	-1236.225382	-555.3127179	-1791.5381
TCGA-V4-A9ED-01	-516.1946528	-176.7985886	-692.9932414
TCGA-V3-A9ZX-01	-912.9509515	215.4125071	-697.5384444
TCGA-VD-A8KJ-01	-1387.856382	-1260.64982	-2648.506202
TCGA-YZ-A985-01	-1240.549834	-935.9068414	-2176.456675
TCGA-V4-A9EJ-01	-1456.770539	-985.9915014	-2442.76204
TCGA-VD-A8KE-01	-1172.298107	-988.026107	-2160.324214
TCGA-V4-A9ES-01	-1763.107171	-1221.944383	-2985.051554
TCGA-WC-A88A-01	-1039.640196	-881.2391226	-1920.879319
TCGA-V4-A9EH-01	-1214.093356	-323.6078516	-1537.701207
TCGA-WC-A87Y-01	-1042.052548	8.304155502	-1033.748392
TCGA-V4-A9F0-01	-696.7340407	-381.1058187	-1077.839859
TCGA-V4-A9EM-01	-1089.331361	-803.3857222	-1892.717084
TCGA-V4-A9EO-01	-1346.060444	-1355.704478	-2701.764922
TCGA-WC-A888-01	-885.6994454	1055.129329	169.4298838
TCGA-VD-A8KD-01	-840.0975812	893.6357166	53.53813538
TCGA-V4-A9ET-01	-1348.751742	-1140.868541	-2489.620283
TCGA-V4-A9EC-01	-1465.003532	-1203.847871	-2668.851403
TCGA-V4-A9EQ-01	-961.386999	-461.5677101	-1422.954709
TCGA-VD-A8KH-01	-1711.789992	-1397.028368	-3108.818359
TCGA-V4-A9F5-01	-1484.988661	-1104.115063	-2589.103725
TCGA-WC-A883-01	-1062.728823	-965.9880957	-2028.716918
TCGA-WC-AA9A-01	-1851.237963	-1471.790882	-3323.028846
TCGA-VD-A8KF-01	-1163.913414	-854.8878053	-2018.801219
TCGA-VD-A8KG-01	-1113.003497	-436.0056268	-1549.009124

TCGA-V4-A9EL-01	-782.3911633	535.101705	-247.2894584
TCGA-V4-A9E8-01	-572.8840561	441.3230938	-131.5609623
TCGA-V4-A9F3-01	-677.5231799	514.9835843	-162.5395956
TCGA-WC-A885-01	-1777.925574	-1404.888014	-3182.813588
TCGA-V4-A9EK-01	-1060.714818	-77.61599499	-1138.330813
TCGA-V4-A9EU-01	-1149.015525	-519.2707925	-1668.286318
TCGA-RZ-AB0B-01	-1146.971909	-890.6633148	-2037.635224
TCGA-V4-A9EX-01	-1259.915276	-927.3432104	-2187.258486
TCGA-WC-A87W-01	-929.2991372	-1377.462331	-2306.761469
TCGA-WC-A87U-01	-1378.554365	-1166.132009	-2544.686374
TCGA-VD-A8KK-01	-1168.54051	-659.4917431	-1828.032253
TCGA-V4-A9EV-01	-729.6063709	1504.811999	775.2056278
TCGA-VD-A8KO-01	-1712.443194	-1505.595047	-3218.038241
TCGA-VD-A8KN-01	-688.5302495	276.9553022	-411.5749474
TCGA-V4-A9EW-01	-1484.314208	-1329.392747	-2813.706955
TCGA-V4-A9E5-01	-1346.455615	-926.4395548	-2272.89517
TCGA-V4-A9F7-01	-1666.433053	-1312.792268	-2979.225321
TCGA-V4-A9EA-01	-1309.41108	-912.0443658	-2221.455446
TCGA-WC-A884-01	-521.2548401	-149.964478	-671.2193181
TCGA-VD-A8KB-01	-1443.392536	-1537.493193	-2980.88573
TCGA-YZ-A982-01	-1296.743205	-603.4258051	-1900.16901
TCGA-YZ-A984-01	-884.2250894	-1273.092137	-2157.317227
TCGA-VD-A8K8-01	-1251.125105	391.0137783	-860.1113263
TCGA-YZ-A983-01	-1490.733861	-925.4379616	-2416.171823
TCGA-V3-A9ZY-01	-1280.339045	-743.8115802	-2024.150625
TCGA-V4-A9EE-01	-1111.630217	-351.1701049	-1462.800322
TCGA-VD-A8KA-01	-1557.436164	-1370.682124	-2928.118288
TCGA-V4-A9F2-01	-740.0534428	-229.2675581	-969.3210009
TCGA-V4-A9EF-01	-1169.294263	-840.0438503	-2009.338113
TCGA-V4-A9E7-01	-1717.736288	-1373.278038	-3091.014326
TCGA-VD-A8K7-01	-1634.651593	-1415.342741	-3049.994334
TCGA-VD-AA8R-01	-1501.019626	-1416.255195	-2917.27482
TCGA-VD-AA8S-01	-770.5917738	-812.8210978	-1583.412872
