# Supplementary material for: Identification of prognostic genes in uveal melanoma microenvironment
Source: PLoS One. 2020 Nov 16;15(11):e0242263. doi: 10.1371/journal.pone.0242263 (PMC7668584; doi:10.1371/journal.pone.0242263)
Supplement: S2 Table — (PDF) [file pone.0242263.s002.pdf]

**S2 Table. 959 intersection differentially expressed genes between high and low scores.**

| Gene | Regulation |
|---|---|
| ASS1 | up |
| SPN | up |
| LRRTM2 | up |
| ITGA4 | up |
| ITGA5 | up |
| SDK2 | up |
| CHST1 | up |
| ITGAX | up |
| BCL2A1 | up |
| ITGAL | up |
| NOV | up |
| COX4I2 | up |
| CRTAM | up |
| ATP1A3 | up |
| ATP1A2 | up |
| IL10RA | up |
| CFB | up |
| CFP | up |
| LOC283070 | up |
| RUNX2 | up |
| IGFBP2 | up |
| IGFBP4 | up |
| APLNR | up |
| CLIC2 | up |
| CLIC3 | up |
| FOXL1 | up |
| HLA-DPA1 | up |
| ZAP70 | up |
| LILRB3 | up |
| NDRG4 | up |
| HLA-DQB1 | up |
| MRC2 | up |
| FOXS1 | up |
| LPAR5 | up |
| MYO1G | up |
| MYO1F | up |
| GALNTL4 | up |
| LGALS9 | up |
| LGALS3 | up |
| LGALS2 | up |
| ASAP3 | up |
| ITGAD | up |
| LOC96610 | up |
| ARHGAP25 | up |
| C1orf38 | up |
| RSAD2 | up |
| CAPG | up |

| | |
|---|---|
| FCER1G | up |
| SLA2 | up |
| FAM49A | up |
| TNFSF10 | up |
| EVI2A | up |
| EVI2B | up |
| GNA15 | up |
| GRIN2D | up |
| BCAT1 | up |
| NOVA1 | up |
| CCL21 | up |
| CCL22 | up |
| CCL26 | up |
| FAM70A | up |
| FAM70B | up |
| UBE2L6 | up |
| ZNF831 | up |
| CD200R1 | up |
| RARRES2 | up |
| IL2RA | up |
| IL2RB | up |
| IL2RG | up |
| RARRES3 | up |
| C16orf54 | up |
| PRKCDBP | up |
| P2RY8 | up |
| MMP25 | up |
| LIF | up |
| NCF1 | up |
| HLA-DRB1 | up |
| HLA-DRB6 | up |
| XAF1 | up |
| HLA-DRB5 | up |
| FN1 | up |
| SYT5 | up |
| LAG3 | up |
| SASH3 | up |
| GAL3ST4 | up |
| HLA-DMB | up |
| TBX21 | up |
| PLA2G7 | up |
| FPR1 | up |
| FPR3 | up |
| NT5E | up |
| LGALS9C | up |
| MAP1A | up |
| MAP1B | up |
| VGF | up |
| TMEM200B | up |

| | |
|---|---|
| TMEM156 | up |
| C1S | up |
| C1R | up |
| PRF1 | up |
| TMEM178 | up |
| CD300C | up |
| EOMES | up |
| KIAA0748 | up |
| ICOS | up |
| ADAM11 | up |
| ADAM19 | up |
| CPNE5 | up |
| UCP2 | up |
| PCSK2 | up |
| SLC16A6 | up |
| SCRG1 | up |
| OSR2 | up |
| ISM1 | up |
| CDKN1A | up |
| MFNG | up |
| GJA1 | up |
| GJA5 | up |
| VCAM1 | up |
| PLEKHO1 | up |
| TIGIT | up |
| IFITM1 | up |
| POU2AF1 | up |
| TM4SF19 | up |
| LAMB3 | up |
| CYSLTR2 | up |
| GPR162 | up |
| TGFB1 | up |
| COX7A1 | up |
| DLL4 | up |
| IFI6 | up |
| STAB1 | up |
| ABI3 | up |
| RHOH | up |
| IL12RB1 | up |
| ADCY1 | up |
| ADCY2 | up |
| PDLIM4 | up |
| CLEC7A | up |
| CTLA4 | up |
| KCNA3 | up |
| IL15 | up |
| PLA2G2D | up |
| HS3ST2 | up |
| CYFIP2 | up |

| | |
|---|---|
| BCL3 | up |
| OAS2 | up |
| TRIM22 | up |
| OASL | up |
| ATP8B4 | up |
| DES | up |
| FBXL7 | up |
| HTR2B | up |
| TLR8 | up |
| ACE | up |
| HLA-C | up |
| SLC7A7 | up |
| GLIPR2 | up |
| GLIPR1 | up |
| GGT1 | up |
| PTPRC | up |
| PTPRO | up |
| SIRPG | up |
| ABLIM3 | up |
| MX2 | up |
| P2RY10 | up |
| ISG15 | up |
| GGTLC2 | up |
| CRIP1 | up |
| SIGLEC1 | up |
| SIPA1L2 | up |
| SLC15A3 | up |
| KLRB1 | up |
| ALDH1L2 | up |
| IRF1 | up |
| IRF5 | up |
| IRF8 | up |
| CSF2RB | up |
| PANX2 | up |
| PTPRE | up |
| ADAP2 | up |
| GAB3 | up |
| ITK | up |
| NPTX1 | up |
| CYBB | up |
| HLA-DQB2 | up |
| ROR2 | up |
| SPATA18 | up |
| GLDC | up |
| FCN1 | up |
| CR1 | up |
| TNFRSF1B | up |
| PTPN7 | up |
| PTPN6 | up |

| | |
|---|---|
| S100A4 | up |
| FOXF1 | up |
| SERPINA1 | up |
| CD69 | up |
| CHRD | up |
| GPR55 | up |
| PAEP | up |
| HSPB8 | up |
| NCRNA00152 | up |
| IL21R | up |
| FAM43B | up |
| CMPK2 | up |
| HPCA | up |
| RFTN1 | up |
| HTATIP2 | up |
| KCTD17 | up |
| TNFSF13B | up |
| GLIS3 | up |
| CYP2S1 | up |
| GPBAR1 | up |
| ELMO1 | up |
| AIF1 | up |
| PDCD1 | up |
| KLRD1 | up |
| SLC29A4 | up |
| GPC4 | up |
| HEPH | up |
| GPR183 | up |
| PIK3CG | up |
| LOC541471 | up |
| RPS6KA2 | up |
| PTPRCAP | up |
| PATL2 | up |
| LRRC25 | up |
| ARL11 | up |
| CLIP3 | up |
| MNDA | up |
| CD48 | up |
| CA12 | up |
| MFSD2A | up |
| C10orf54 | up |
| NLRP1 | up |
| CSF1R | up |
| CDCP1 | up |
| XCL2 | up |
| FCGR2B | up |
| CYTH4 | up |
| PTGS1 | up |
| ACSM5 | up |

| | |
|---|---|
| CDH23 | up |
| FLRT1 | up |
| ARHGAP9 | up |
| SLA | up |
| MGC12982 | up |
| GMFG | up |
| IDO1 | up |
| CAMK1D | up |
| ECM1 | up |
| LYPD1 | up |
| RGS1 | up |
| LOXL3 | up |
| SECTM1 | up |
| CD27 | up |
| IFI30 | up |
| PLA2R1 | up |
| HCST | up |
| HTRA1 | up |
| CCDC109B | up |
| MAPK10 | up |
| FZD2 | up |
| RASSF5 | up |
| TOX2 | up |
| CSF3R | up |
| CXCL11 | up |
| CXCL10 | up |
| CXCL13 | up |
| CXCL12 | up |
| TNFRSF4 | up |
| FIBCD1 | up |
| TMSL3 | up |
| IL1RN | up |
| PRAM1 | up |
| AK5 | up |
| NCKAP1L | up |
| CXCR2P1 | up |
| AOAH | up |
| C3AR1 | up |
| SCN1B | up |
| TSC22D3 | up |
| PLCE1 | up |
| PTGER2 | up |
| SMOC2 | up |
| CYP1B1 | up |
| S1PR4 | up |
| TYMP | up |
| CTSS | up |
| CTSW | up |
| CXCL9 | up |

| | |
|---|---|
| EMB | up |
| CARD9 | up |
| ADAMTS2 | up |
| GPR65 | up |
| GPR68 | up |
| TMEM37 | up |
| SDS | up |
| SIGLEC12 | up |
| KCNK6 | up |
| AOC3 | up |
| LYZ | up |
| SUSD4 | up |
| LY9 | up |
| TNIK | up |
| HLA-B | up |
| HLA-F | up |
| PSTPIP1 | up |
| TFEC | up |
| VAV1 | up |
| TNFAIP2 | up |
| PRR5L | up |
| C5orf20 | up |
| GFI1 | up |
| TAP1 | up |
| AMZ1 | up |
| SLITRK2 | up |
| SGK1 | up |
| LST1 | up |
| GDF11 | up |
| GZMA | up |
| GZMB | up |
| GZMM | up |
| GZMH | up |
| GZMK | up |
| LEF1 | up |
| B4GALNT1 | up |
| ASIP | up |
| CA8 | up |
| C12orf68 | up |
| FUT7 | up |
| SIGLEC14 | up |
| SIGLEC11 | up |
| SPI1 | up |
| CCL4L2 | up |
| SLAMF6 | up |
| SLAMF1 | up |
| F13A1 | up |
| C6orf105 | up |
| FCRL3 | up |

| | |
|---|---|
| FCRL6 | up |
| MGAT3 | up |
| MMP9 | up |
| MMP2 | up |
| AMICA1 | up |
| DOCK2 | up |
| ACSBG1 | up |
| SLAMF8 | up |
| GIMAP8 | up |
| GIMAP4 | up |
| GIMAP5 | up |
| GIMAP6 | up |
| GIMAP7 | up |
| GIMAP1 | up |
| WARS | up |
| LAX1 | up |
| CTNND2 | up |
| ZMYND15 | up |
| CORO1A | up |
| TRIM9 | up |
| LOC100233209 | up |
| SLC47A1 | up |
| TSPAN5 | up |
| SNX20 | up |
| FSTL4 | up |
| TLR7 | up |
| FCRL5 | up |
| UBD | up |
| AMN | up |
| MEFV | up |
| RASL11B | up |
| CARD16 | up |
| PDCD1LG2 | up |
| COL9A2 | up |
| COL9A3 | up |
| SEMA6B | up |
| KCNJ10 | up |
| MMP1 | up |
| C17orf76 | up |
| SLC6A1 | up |
| PECAM1 | up |
| TRPV2 | up |
| COL22A1 | up |
| SLC4A8 | up |
| CIITA | up |
| LSP1 | up |
| TOX | up |
| HMOX1 | up |
| IGSF6 | up |

| | |
|---|---|
| APOL1 | up |
| LCP2 | up |
| LCP1 | up |
| SULF2 | up |
| RASAL3 | up |
| FGR | up |
| COL5A3 | up |
| COL5A1 | up |
| BAI2 | up |
| HCK | up |
| HLA-DPB1 | up |
| CHAC1 | up |
| ITGA11 | up |
| PARP8 | up |
| DPYS | up |
| TUBB3 | up |
| PSMB9 | up |
| SLCO2B1 | up |
| ADRA2A | up |
| CCR1 | up |
| CCR2 | up |
| CCR5 | up |
| CCR7 | up |
| CD84 | up |
| CD86 | up |
| ZBED2 | up |
| CD8A | up |
| CD8B | up |
| RUNX3 | up |
| HLA-DOA | up |
| HLA-DOB | up |
| CCDC74A | up |
| PTPN22 | up |
| SPOCD1 | up |
| NFAM1 | up |
| HLA-DQA1 | up |
| HLA-DQA2 | up |
| SERPINB8 | up |
| HK3 | up |
| MLC1 | up |
| TNF | up |
| BATF | up |
| PIK3R5 | up |
| XCL1 | up |
| TNFAIP8L2 | up |
| REEP2 | up |
| CECR1 | up |
| CHRNA1 | up |
| ID3 | up |

| | |
|---|---|
| UBA7 | up |
| SIGLEC8 | up |
| CD5L | up |
| NBL1 | up |
| SIGLEC7 | up |
| FOLR2 | up |
| LAPTM5 | up |
| B2M | up |
| SIGLEC5 | up |
| LILRB5 | up |
| LILRB2 | up |
| LILRB1 | up |
| GRAP2 | up |
| C9orf110 | up |
| BIN2 | up |
| DPYD | up |
| HLA-DMA | up |
| RHBDL2 | up |
| CD300A | up |
| MDGA1 | up |
| MYCN | up |
| IGF2 | up |
| IGF1 | up |
| FLJ42875 | up |
| CD300LF | up |
| CD300LB | up |
| PRIMA1 | up |
| NINJ2 | up |
| ARHGAP30 | up |
| CMKLR1 | up |
| PTAFR | up |
| ATP6V0D2 | up |
| PRDM1 | up |
| PPM1K | up |
| PLD4 | up |
| NNMT | up |
| SUSD2 | up |
| PILRA | up |
| C3 | up |
| KCNJ5 | up |
| KCNJ4 | up |
| AGPAT4 | up |
| COL1A1 | up |
| RCSD1 | up |
| FLI1 | up |
| C19orf35 | up |
| KLHL6 | up |
| BLNK | up |
| KCNMB4 | up |

| | |
|---|---|
| SOCS2 | up |
| TARP | up |
| CCNO | up |
| CCL3L1 | up |
| IL1R2 | up |
| ITPR2 | up |
| CES1 | up |
| ACAN | up |
| FFAR3 | up |
| DAPP1 | up |
| P2RX1 | up |
| SPNS3 | up |
| IFNG | up |
| ARHGAP15 | up |
| IL7R | up |
| FGL2 | up |
| ME1 | up |
| LRMP | up |
| PGCP | up |
| SLIT1 | up |
| CCL3 | up |
| NFATC2 | up |
| NFATC4 | up |
| CCL4 | up |
| CCL8 | up |
| GCET2 | up |
| MGLL | up |
| RASGRF1 | up |
| TRPM2 | up |
| GPR132 | up |
| TBXAS1 | up |
| INPP5D | up |
| TRAF3IP3 | up |
| TGFBI | up |
| LCK | up |
| IL1B | up |
| LILRB4 | up |
| ADAM23 | up |
| ADAM28 | up |
| IL18 | up |
| GSTM5 | up |
| LTB | up |
| SSX6 | up |
| SSX4 | up |
| SSX1 | up |
| MS4A7 | up |
| MS4A1 | up |
| FASLG | up |
| SLC45A1 | up |

| | |
|---|---|
| CCDC64 | up |
| TMEM119 | up |
| FAM105A | up |
| WWTR1 | up |
| SORCS1 | up |
| CCL13 | up |
| CCL14 | up |
| CCL18 | up |
| CCL19 | up |
| ALOX15B | up |
| NTNG2 | up |
| THY1 | up |
| MMP12 | up |
| HLA-DRA | up |
| PRG2 | up |
| KCNK13 | up |
| SIRPB2 | up |
| P2RY13 | up |
| IFI44L | up |
| LTA | up |
| LTF | up |
| IL32 | up |
| SAMSN1 | up |
| MGC29506 | up |
| EMR1 | up |
| NCF1B | up |
| TMEM130 | up |
| TMEM155 | up |
| AIM1 | up |
| AIM2 | up |
| IL18BP | up |
| NOD2 | up |
| HCLS1 | up |
| RNASE6 | up |
| TUBA4A | up |
| SSX5 | up |
| P2RY6 | up |
| SIGLEC9 | up |
| APBB1IP | up |
| SYT11 | up |
| GEM | up |
| GPR171 | up |
| GPR174 | up |
| GPR176 | up |
| UCHL1 | up |
| SP140 | up |
| HSH2D | up |
| LOC400696 | up |
| CASP1 | up |

| | |
|---|---|
| PRDM8 | up |
| ALOX5 | up |
| TNFRSF10C | up |
| PROCR | up |
| NCF2 | up |
| NCF4 | up |
| CNTNAP1 | up |
| APOB48R | up |
| PAPSS2 | up |
| DPEP2 | up |
| DYSF | up |
| PPP2R2B | up |
| GATA6 | up |
| GATA3 | up |
| SH2D1A | up |
| IPCEF1 | up |
| C4A | up |
| SNX32 | up |
| ANPEP | up |
| LAMA1 | up |
| PLAC8 | up |
| CASS4 | up |
| BAALC | up |
| SLCO5A1 | up |
| APOL6 | up |
| APOL4 | up |
| APOL3 | up |
| C1QC | up |
| C1QB | up |
| C1QA | up |
| TAGAP | up |
| VSIG4 | up |
| ITGB2 | up |
| PLXDC2 | up |
| PLXDC1 | up |
| ST8SIA4 | up |
| CD74 | up |
| CD70 | up |
| NLRP3 | up |
| BTLA | up |
| ASCL2 | up |
| LOC606724 | up |
| CD109 | up |
| CD247 | up |
| LILRA5 | up |
| APOC2 | up |
| SELL | up |
| BST2 | up |
| DOCK11 | up |

| | |
|---|---|
| GPR37L1 | up |
| C1orf162 | up |
| NGFR | up |
| MCHR1 | up |
| EPSTI1 | up |
| GFRA2 | up |
| FAM113B | up |
| ZNF385A | up |
| ISG20 | up |
| CD52 | up |
| CD53 | up |
| KIAA1324 | up |
| RAB31 | up |
| DOK2 | up |
| CD163L1 | up |
| ARMC9 | up |
| CD163 | up |
| IQSEC3 | up |
| DNASE1L3 | up |
| CD40LG | up |
| TUBB2B | up |
| MYEOV | up |
| SLIT3 | up |
| ZNF683 | up |
| SEL1L3 | up |
| NIPAL4 | up |
| CCRL2 | up |
| CHI3L2 | up |
| CHI3L1 | up |
| THEMIS | up |
| ODZ4 | up |
| ASB2 | up |
| GPR114 | up |
| F2RL3 | up |
| CCL5 | up |
| LAMP3 | up |
| CD38 | up |
| IFI27 | up |
| CD33 | up |
| CD37 | up |
| PSD2 | up |
| TMEM176A | up |
| TMEM176B | up |
| CD3D | up |
| CD3E | up |
| CD3G | up |
| CD209 | up |
| LAIR1 | up |
| HCP5 | up |

| | |
|---|---|
| ADAMTS14 | up |
| IGSF21 | up |
| RASGRP1 | up |
| FCGR3A | up |
| ALDH1A1 | up |
| LOC400759 | up |
| CALHM2 | up |
| FAM26F | up |
| HAVCR2 | up |
| FXYD2 | up |
| ADAM6 | up |
| OLFML3 | up |
| ANKRD22 | up |
| CYTIP | up |
| TNFRSF9 | up |
| S100A9 | up |
| FHDC1 | up |
| TBC1D10C | up |
| ABP1 | up |
| TM7SF4 | up |
| CD14 | up |
| GGTA1 | up |
| GADD45G | up |
| RIMS2 | up |
| GRID1 | up |
| MSR1 | up |
| CERKL | up |
| BATF3 | up |
| KLHDC7B | up |
| KLRK1 | up |
| SLC1A3 | up |
| LRRC10B | up |
| FCGR1B | up |
| FCGR1A | up |
| DHRS9 | up |
| DHRS3 | up |
| JAKMIP1 | up |
| GNLY | up |
| IL15RA | up |
| SLC2A3 | up |
| SLC46A3 | up |
| LAT2 | up |
| S1PR5 | up |
| COBL | up |
| TNFSF8 | up |
| NUDT16P1 | up |
| MS4A6A | up |
| SCML4 | up |
| PKD2L1 | up |

| | |
|---|---|
| FMO3 | up |
| GBP1 | up |
| FRZB | up |
| ST8SIA1 | up |
| CD180 | up |
| WAS | up |
| DPP4 | up |
| A4GALT | up |
| MPEG1 | up |
| ADAMDEC1 | up |
| CD7 | up |
| SDC2 | up |
| ELN | up |
| SLC8A1 | up |
| C12orf75 | up |
| PTGIR | up |
| AHNAK2 | up |
| SQRDL | up |
| SCN4B | up |
| MS4A4A | up |
| ACTA2 | up |
| TSTD1 | up |
| PLN | up |
| PTGER4 | up |
| KIR2DL4 | up |
| NCF1C | up |
| HTRA3 | up |
| C17orf87 | up |
| SPIB | up |
| SLC38A5 | up |
| RASGRP2 | up |
| NLRC5 | up |
| SLC38A3 | up |
| CXCR3 | up |
| CXCR6 | up |
| CXCR5 | up |
| IFI44 | up |
| TREM2 | up |
| THBS2 | up |
| CADM1 | up |
| FGD2 | up |
| CELF2 | up |
| PTGDS | up |
| NKG7 | up |
| CARD11 | up |
| HCG26 | up |
| RIPK3 | up |
| C20orf103 | up |
| GPR34 | up |

| | |
|---|---|
| AQP3 | up |
| AQP1 | up |
| ADORA3 | up |
| EPHB2 | up |
| EPHB1 | up |
| TIFAB | up |
| WDFY4 | up |
| C17orf60 | up |
| DARC | up |
| PHYHIP | up |
| CLEC12A | up |
| PLEK | up |
| SPOCK2 | up |
| GAD1 | up |
| TMEM150B | up |
| FEZ1 | up |
| KIF21B | up |
| BCL11B | up |
| PALM3 | up |
| UBASH3A | up |
| C21orf121 | up |
| MAN1C1 | up |
| MYO7A | up |
| NAPSB | up |
| TYROBP | up |
| GPR109A | up |
| SAMD3 | up |
| EGFLAM | up |
| GPR18 | up |
| KCNMA1 | up |
| IGJ | up |
| EBI3 | up |
| SIT1 | up |
| PARVG | up |
| CXorf21 | up |
| PLEKHG4B | up |
| SELPLG | up |
| SPON1 | up |
| TIMP4 | up |
| DKK3 | up |
| LILRA6 | up |
| LILRA4 | up |
| LILRA2 | up |
| ZNF467 | up |
| HPSE | up |
| OSCAR | up |
| SORL1 | up |
| CD96 | up |
| CLEC10A | up |

| | |
|---|---|
| CST7 | up |
| SKAP1 | up |
| CSTA | up |
| AMIGO2 | up |
| SLC6A9 | up |
| TRAT1 | up |
| LINGO1 | up |
| TNFRSF8 | up |
| FCGR1C | up |
| RGS18 | up |
| LY86 | up |
| CRHBP | up |
| ZBP1 | up |
| MALL | up |
| GBP2 | up |
| GBP5 | up |
| GBP4 | up |
| CORO2B | up |
| CD4 | up |
| CD5 | up |
| CD6 | up |
| CD2 | up |
| STAT1 | up |
| VCAN | up |
| ACY3 | up |
| SIGLEC10 | up |
| ARC | up |
| ABCC3 | up |
| APLP1 | up |
| GLI2 | up |
| MAFB | up |
| SLCO3A1 | up |
| SLC2A6 | up |
| C9orf109 | up |
| PLAU | up |
| ETV7 | up |
| IKZF1 | up |
| IKZF3 | up |
| IL4I1 | up |
| FOXD2 | up |
| ACAP1 | up |
| GNGT2 | up |
| C4orf19 | down |
| ZNF285 | down |
| PCDH20 | down |
| TMEM84 | down |
| MPZ | down |
| CCL28 | down |
| CTF1 | down |

| | |
|---|---|
| FBXO17 | down |
| KIAA1543 | down |
| ADRA2C | down |
| HFE | down |
| CES3 | down |
| TMEM40 | down |
| GNAL | down |
| ZNF727 | down |
| FAM132A | down |
| EDNRB | down |
| ZNF114 | down |
| MUC7 | down |
| IL12RB2 | down |
| TRIM58 | down |
| HILS1 | down |
| LMCD1 | down |
| ZNF676 | down |
| ALDH1L1 | down |
| C13orf35 | down |
| ACSM3 | down |
| PERP | down |
| ZNF229 | down |
| SLC3A1 | down |
| CKMT1B | down |
| C6orf142 | down |
| ZNF793 | down |
| PDE3A | down |
| KCNK2 | down |
| FAM75A2 | down |
| NR6A1 | down |
| FUT3 | down |
| GSTA3 | down |
| DMRT2 | down |
| ZNF826 | down |
| SLC44A3 | down |
| ZNF257 | down |
| TDRD5 | down |
| ASB10 | down |
| SERPINB9 | down |
| CHGB | down |
| PPAN-P2RY11 | down |
| ABCA17P | down |
| EPCAM | down |
| GPR27 | down |
| RNF43 | down |
| ZNF883 | down |
| MTUS1 | down |
| GSTO2 | down |
| ERBB3 | down |

| | |
|---|---|
| GJB1 | down |
| ZNF560 | down |
| ZNF541 | down |
| ZNF677 | down |
| NEDD9 | down |
| ZNF391 | down |
| LOC100190938 | down |
| PCOLCE2 | down |
| SLC25A21 | down |
| HOMER2 | down |
| ZNF681 | down |
| AZGP1 | down |
| WNK4 | down |
| LOC100270746 | down |
| KREMEN2 | down |
| RAPGEF3 | down |
| ZNF208 | down |
| ZSCAN23 | down |
| LIPH | down |
| ZNF781 | down |
| ZNF415 | down |
| MYH7 | down |
| CELF5 | down |
| ZNF667 | down |
| STEAP1 | down |
| LINGO2 | down |
| ACSF2 | down |
| PEG3 | down |
| CPS1 | down |
| PLEKHG4 | down |
