# Supplementary material for: Identification of prognostic genes in uveal melanoma microenvironment
Source: PLoS One. 2020 Nov 16;15(11):e0242263. doi: 10.1371/journal.pone.0242263 (PMC7668584; doi:10.1371/journal.pone.0242263)
Supplement: S3 Table — (PDF) [file pone.0242263.s003.pdf]

S3 Table. 475 genes were significantly predicting the prognosis of UM patients in the training cohort by both Kaplan-Meier and univariate Cox regression analyses (p-value < 0.01).

Gene	Kaplan-Meier analysis (p-value)	Univariate Cox regression analysis			
		HR	HR.95L	HR.95H	P-value
ASS1	4.48E-05	2.504	1.513	4.144	3.57E-04
SPN	3.36E-05	1.432	1.165	1.762	6.69E-04
LRRTM2	1.40E-05	1.398	1.157	1.690	5.28E-04
ITGA4	6.82E-03	1.295	1.084	1.547	4.34E-03
ITGA5	5.38E-07	3.063	1.869	5.021	9.02E-06
SDK2	2.05E-04	1.523	1.191	1.947	8.10E-04
ITGAX	2.94E-04	1.779	1.342	2.359	6.14E-05
ITGAL	2.00E-03	1.405	1.166	1.692	3.55E-04
NOV	4.43E-03	1.611	1.201	2.162	1.45E-03
COX4I2	9.19E-03	1.567	1.194	2.056	1.19E-03
CRTAM	5.59E-03	1.383	1.133	1.687	1.40E-03
IL10RA	2.20E-03	1.490	1.135	1.956	4.07E-03
LOC283070	6.13E-04	1.352	1.084	1.687	7.39E-03
C4orf19	1.39E-03	0.671	0.530	0.849	9.19E-04
ZNF285	3.01E-03	0.666	0.529	0.839	5.71E-04
RUNX2	7.44E-03	1.703	1.204	2.408	2.60E-03
IGFBP2	4.08E-03	2.116	1.428	3.137	1.89E-04
FOXL1	2.65E-03	2.016	1.359	2.990	4.90E-04
HLA-DPA1	4.80E-04	1.491	1.154	1.927	2.22E-03
ZAP70	1.93E-03	1.364	1.142	1.630	6.09E-04
PCDH20	3.04E-03	0.765	0.662	0.885	3.20E-04
HLA-DQB1	8.85E-03	1.324	1.083	1.620	6.30E-03
MRC2	6.24E-05	1.692	1.281	2.236	2.14E-04
FOXS1	8.91E-03	1.782	1.222	2.597	2.67E-03
MYO1G	1.68E-04	1.876	1.415	2.486	1.22E-05
MYO1F	1.78E-03	1.755	1.219	2.526	2.49E-03
GALNTL4	1.56E-03	1.675	1.241	2.259	7.36E-04
LGALS3	8.67E-03	1.449	1.118	1.879	5.12E-03
LGALS2	5.64E-03	1.391	1.125	1.720	2.34E-03

LOC96610	6.89E-03	1.370	1.137	1.650	9.28E-04
C1orf38	1.02E-04	2.631	1.676	4.131	2.64E-05
MPZ	2.72E-07	0.586	0.464	0.740	7.36E-06
SLA2	2.34E-04	1.432	1.187	1.726	1.73E-04
EVI2B	2.53E-04	1.449	1.120	1.875	4.77E-03
GRIN2D	2.99E-03	1.811	1.261	2.601	1.29E-03
BCAT1	1.45E-05	1.606	1.277	2.019	5.01E-05
NOVA1	1.49E-04	1.434	1.162	1.771	8.02E-04
CCL28	8.26E-03	0.519	0.336	0.802	3.10E-03
FAM70A	1.24E-03	1.522	1.237	1.873	7.31E-05
FAM70B	2.14E-03	1.623	1.195	2.204	1.93E-03
CTF1	7.28E-07	0.578	0.463	0.721	1.27E-06
IL2RB	2.23E-04	1.418	1.155	1.739	8.22E-04
IL2RG	2.05E-03	1.411	1.173	1.698	2.69E-04
C16orf54	4.05E-04	1.400	1.132	1.732	1.93E-03
P2RY8	6.77E-03	2.107	1.393	3.186	4.14E-04
FBXO17	5.01E-07	0.609	0.477	0.777	6.91E-05
LIF	2.00E-03	1.455	1.152	1.838	1.64E-03
KIAA1543	1.54E-07	0.624	0.506	0.770	1.10E-05
ADRA2C	7.27E-03	0.628	0.476	0.828	9.82E-04
HLA-DRB1	8.41E-03	1.411	1.131	1.760	2.30E-03
FN1	5.02E-03	1.873	1.324	2.649	3.88E-04
SYT5	4.83E-05	1.291	1.072	1.555	6.96E-03
HFE	4.73E-05	0.584	0.456	0.748	2.11E-05
LAG3	4.77E-05	1.307	1.144	1.492	8.18E-05
NT5E	1.75E-03	1.302	1.081	1.567	5.40E-03
TMEM40	7.23E-03	0.567	0.385	0.836	4.15E-03
MAP1A	2.30E-05	1.395	1.154	1.687	5.91E-04
VGF	8.08E-06	1.405	1.212	1.628	6.66E-06
C1S	7.11E-03	1.418	1.129	1.782	2.66E-03
PRF1	2.98E-06	1.430	1.188	1.720	1.53E-04
EOMES	5.79E-03	1.364	1.130	1.646	1.25E-03
ICOS	2.07E-03	1.395	1.154	1.687	5.85E-04

ADAM11	1.33E-05	1.897	1.412	2.549	2.14E-05
ADAM19	1.08E-03	1.397	1.135	1.721	1.63E-03
SLC16A6	6.71E-05	1.611	1.249	2.076	2.34E-04
OSR2	7.71E-06	1.641	1.294	2.080	4.35E-05
ISM1	4.53E-06	1.522	1.264	1.834	9.80E-06
MFNG	1.07E-03	1.827	1.209	2.760	4.24E-03
EDNRB	3.53E-04	0.478	0.347	0.657	5.42E-06
GJA5	6.11E-03	1.643	1.225	2.203	9.20E-04
PLEKHO1	3.38E-03	1.963	1.336	2.886	5.99E-04
TIGIT	4.87E-05	1.330	1.140	1.551	2.84E-04
TM4SF19	5.58E-03	1.621	1.238	2.122	4.44E-04
LAMB3	1.88E-03	1.314	1.107	1.560	1.82E-03
GPR162	8.82E-07	1.321	1.091	1.598	4.27E-03
COX7A1	1.30E-03	1.585	1.185	2.119	1.91E-03
DLL4	8.47E-08	2.252	1.566	3.238	1.18E-05
RHOH	2.34E-04	1.399	1.150	1.701	7.73E-04
IL12RB1	4.55E-03	1.362	1.135	1.634	8.93E-04
IL12RB2	1.17E-06	0.742	0.636	0.865	1.43E-04
ADCY1	7.55E-05	1.327	1.131	1.558	5.19E-04
ADCY2	8.95E-04	1.300	1.098	1.539	2.32E-03
PDLIM4	5.51E-03	1.395	1.117	1.743	3.38E-03
BCL3	5.84E-05	2.697	1.637	4.442	9.76E-05
FBXL7	3.81E-03	1.666	1.208	2.297	1.85E-03
HTR2B	2.73E-04	1.358	1.174	1.570	3.58E-05
TLR8	2.20E-04	1.426	1.130	1.800	2.79E-03
HLA-C	6.57E-03	1.594	1.204	2.109	1.12E-03
SLC7A7	1.23E-03	2.146	1.441	3.195	1.70E-04
HILS1	1.19E-04	0.668	0.528	0.844	7.13E-04
GLIPR2	4.73E-03	2.216	1.358	3.616	1.45E-03
GGT1	3.15E-06	1.671	1.316	2.121	2.48E-05
PTPRC	3.00E-03	1.332	1.114	1.592	1.64E-03
SIRPG	5.86E-04	1.306	1.134	1.505	2.21E-04
P2RY10	5.33E-03	1.400	1.142	1.717	1.20E-03

ISG15	8.91E-04	1.340	1.093	1.643	4.79E-03
GGTLC2	1.84E-08	1.708	1.328	2.197	3.05E-05
CRIP1	5.28E-05	1.515	1.197	1.918	5.56E-04
SIPA1L2	9.57E-05	1.809	1.297	2.524	4.85E-04
LMCD1	1.99E-07	0.507	0.390	0.659	4.09E-07
ZNF676	2.38E-03	0.638	0.484	0.842	1.47E-03
ALDH1L1	1.09E-05	0.632	0.504	0.792	6.61E-05
IRF1	1.83E-04	1.488	1.225	1.807	6.23E-05
IRF5	2.13E-04	3.126	1.772	5.516	8.34E-05
IRF8	4.74E-03	1.461	1.152	1.853	1.76E-03
CSF2RB	2.49E-04	1.532	1.137	2.065	5.04E-03
PANX2	9.72E-05	1.689	1.286	2.220	1.68E-04
PTPRE	7.26E-04	1.689	1.243	2.293	7.95E-04
GAB3	2.03E-04	2.146	1.472	3.130	7.29E-05
NPTX1	2.32E-03	1.307	1.112	1.536	1.17E-03
TNFRSF1B	1.02E-03	1.816	1.320	2.497	2.43E-04
PTPN7	4.56E-04	1.411	1.175	1.695	2.27E-04
S100A4	2.62E-04	1.699	1.362	2.121	2.70E-06
FOXF1	2.34E-03	1.754	1.360	2.262	1.51E-05
CHRD	9.08E-04	1.916	1.312	2.798	7.68E-04
GPR55	5.24E-04	1.516	1.192	1.929	7.03E-04
PAEP	1.60E-03	1.327	1.123	1.568	9.12E-04
NCRNA00152	1.44E-06	1.824	1.402	2.373	7.48E-06
IL21R	4.58E-03	1.392	1.150	1.686	7.06E-04
CMPK2	1.11E-04	1.432	1.133	1.809	2.63E-03
RFTN1	3.25E-04	1.816	1.262	2.613	1.31E-03
HTATIP2	6.71E-07	1.950	1.407	2.703	6.02E-05
KCTD17	6.68E-06	2.509	1.546	4.072	1.97E-04
TNFSF13B	6.58E-03	1.433	1.157	1.774	9.63E-04
GLIS3	1.62E-03	1.682	1.251	2.262	5.71E-04
CYP2S1	3.80E-03	2.012	1.284	3.153	2.30E-03
GPBAR1	6.70E-04	1.553	1.143	2.111	4.93E-03
ELMO1	9.07E-05	1.601	1.187	2.159	2.03E-03

AIF1	3.10E-04	1.577	1.142	2.178	5.62E-03
PDCD1	8.56E-06	1.352	1.158	1.578	1.33E-04
SLC29A4	3.10E-03	1.705	1.214	2.396	2.09E-03
GPC4	1.20E-05	1.716	1.309	2.248	8.98E-05
LOC541471	4.24E-06	1.832	1.370	2.451	4.54E-05
RPS6KA2	1.50E-03	1.668	1.233	2.258	9.11E-04
CD48	1.45E-03	1.423	1.152	1.759	1.07E-03
CA12	2.53E-06	1.558	1.341	1.811	7.05E-09
MFSD2A	2.78E-04	1.367	1.097	1.703	5.42E-03
ARHGAP9	6.04E-03	1.482	1.162	1.890	1.51E-03
ZNF229	7.20E-05	0.716	0.598	0.857	2.79E-04
SLC3A1	3.90E-03	0.693	0.550	0.872	1.79E-03
GMFG	4.49E-04	1.901	1.358	2.662	1.80E-04
IDO1	1.42E-04	1.256	1.106	1.427	4.51E-04
CAMK1D	1.16E-03	1.342	1.101	1.636	3.59E-03
ECM1	3.75E-04	1.622	1.295	2.032	2.58E-05
CD27	6.36E-05	1.413	1.183	1.688	1.35E-04
PLA2R1	8.15E-04	1.465	1.175	1.828	7.11E-04
HTRA1	4.97E-06	1.854	1.351	2.545	1.33E-04
CCDC109B	1.35E-03	1.605	1.212	2.126	9.72E-04
RASSF5	2.49E-05	1.590	1.210	2.090	8.87E-04
CXCL11	7.60E-03	1.363	1.127	1.649	1.41E-03
CXCL10	2.37E-03	1.293	1.115	1.499	6.50E-04
TMSL3	8.89E-03	1.635	1.147	2.331	6.60E-03
AK5	9.32E-03	1.285	1.085	1.521	3.70E-03
CXCR2P1	1.53E-04	1.303	1.129	1.505	2.96E-04
SCN1B	6.49E-06	1.735	1.260	2.388	7.28E-04
TSC22D3	8.24E-04	3.143	1.725	5.728	1.84E-04
C6orf142	1.70E-06	0.715	0.613	0.834	1.92E-05
SMOC2	4.96E-03	1.431	1.104	1.855	6.80E-03
S1PR4	5.67E-03	1.613	1.239	2.100	3.84E-04
TYMP	8.08E-06	1.855	1.375	2.502	5.15E-05
CTSW	1.11E-03	1.398	1.166	1.677	3.02E-04

CXCL9	1.23E-05	1.296	1.139	1.475	8.57E-05
ADAMTS2	3.20E-04	1.474	1.195	1.818	2.95E-04
PDE3A	3.22E-03	0.771	0.657	0.905	1.45E-03
GPR65	7.42E-03	1.475	1.121	1.941	5.55E-03
GPR68	2.36E-03	1.666	1.227	2.264	1.09E-03
SDS	2.26E-03	1.416	1.149	1.747	1.13E-03
SIGLEC12	2.66E-05	1.744	1.343	2.265	2.98E-05
KCNK6	1.99E-03	1.978	1.309	2.990	1.21E-03
KCNK2	3.14E-04	0.704	0.574	0.863	7.42E-04
LYZ	1.22E-03	1.380	1.145	1.664	7.11E-04
LY9	4.31E-05	1.603	1.284	2.000	3.01E-05
HLA-B	9.25E-04	1.491	1.189	1.869	5.30E-04
PSTPIP1	1.21E-04	1.757	1.314	2.348	1.42E-04
FAM75A2	1.71E-04	0.675	0.537	0.849	7.93E-04
TNFAIP2	7.42E-04	1.942	1.383	2.728	1.27E-04
NR6A1	5.57E-06	0.502	0.359	0.701	5.52E-05
PRR5L	3.39E-04	2.050	1.490	2.819	1.03E-05
C5orf20	3.12E-03	1.463	1.188	1.801	3.43E-04
AMZ1	3.06E-06	1.690	1.325	2.156	2.36E-05
SLITRK2	1.28E-04	1.378	1.143	1.661	7.86E-04
SGK1	4.06E-04	1.346	1.074	1.688	9.99E-03
LST1	9.93E-03	1.599	1.191	2.147	1.77E-03
GDF11	1.11E-03	1.915	1.348	2.721	2.88E-04
GZMA	5.28E-05	1.380	1.175	1.622	9.04E-05
GZMB	2.47E-07	1.482	1.236	1.776	2.06E-05
GZMH	9.03E-04	1.522	1.236	1.873	7.41E-05
GZMK	2.66E-04	1.290	1.128	1.476	2.10E-04
LEF1	6.36E-05	1.680	1.272	2.220	2.58E-04
B4GALNT1	2.38E-05	1.440	1.187	1.747	2.20E-04
CA8	6.99E-04	1.352	1.166	1.567	6.29E-05
FUT3	2.95E-04	0.649	0.506	0.833	6.79E-04
GSTA3	9.91E-07	0.731	0.627	0.851	5.64E-05
DMRT2	1.30E-03	0.692	0.568	0.843	2.62E-04

ZNF826	2.35E-03	0.551	0.399	0.760	2.83E-04
CCL4L2	6.63E-05	1.473	1.190	1.823	3.70E-04
SLAMF6	2.26E-03	1.370	1.145	1.638	5.77E-04
SLAMF1	1.41E-03	1.455	1.170	1.810	7.49E-04
FCRL3	7.12E-03	1.281	1.080	1.519	4.42E-03
FCRL6	1.31E-03	1.521	1.218	1.900	2.12E-04
MMP2	9.59E-04	1.466	1.146	1.875	2.31E-03
AMICA1	1.36E-03	1.678	1.259	2.235	4.10E-04
SLAMF8	4.20E-03	1.506	1.194	1.899	5.48E-04
GIMAP8	2.21E-04	1.908	1.255	2.903	2.53E-03
GIMAP4	2.09E-05	1.646	1.229	2.206	8.42E-04
GIMAP6	4.28E-03	1.658	1.184	2.321	3.24E-03
GIMAP1	5.46E-03	1.702	1.204	2.405	2.60E-03
WARS	6.31E-06	1.714	1.348	2.178	1.07E-05
LAX1	5.41E-04	1.459	1.179	1.806	5.13E-04
SLC44A3	1.60E-07	0.467	0.347	0.630	5.80E-07
CORO1A	2.12E-03	1.529	1.171	1.996	1.79E-03
SLC47A1	1.24E-07	2.238	1.532	3.269	3.12E-05
FSTL4	5.42E-04	1.594	1.198	2.120	1.36E-03
UBD	9.99E-03	1.298	1.123	1.499	3.99E-04
AMN	1.55E-04	1.858	1.389	2.485	3.01E-05
RASL11B	2.36E-03	1.495	1.176	1.901	1.02E-03
COL9A3	2.76E-05	1.517	1.239	1.857	5.53E-05
SEMA6B	3.93E-03	1.704	1.225	2.371	1.56E-03
MMP1	6.67E-04	1.515	1.218	1.885	1.90E-04
C17orf76	7.46E-05	2.708	1.715	4.275	1.91E-05
PECAM1	1.18E-03	2.852	1.701	4.782	7.01E-05
TRPV2	2.11E-05	1.762	1.303	2.384	2.39E-04
COL22A1	6.33E-03	1.260	1.089	1.458	1.87E-03
CIITA	3.55E-03	1.349	1.085	1.678	7.08E-03
HMOX1	3.38E-04	2.140	1.500	3.052	2.68E-05
APOL1	1.77E-03	1.319	1.124	1.549	7.14E-04
LCP2	1.03E-05	1.517	1.187	1.939	8.75E-04

LCP1	2.38E-03	1.516	1.181	1.946	1.10E-03
SULF2	9.57E-07	2.184	1.534	3.108	1.45E-05
RASAL3	1.45E-05	1.506	1.206	1.881	3.00E-04
COL5A3	9.79E-03	1.558	1.213	1.999	5.06E-04
BAI2	7.12E-04	1.433	1.160	1.769	8.41E-04
HCK	4.02E-03	1.700	1.231	2.349	1.28E-03
CHAC1	5.74E-07	1.669	1.319	2.112	2.00E-05
PARP8	8.59E-06	2.023	1.460	2.803	2.27E-05
DPYS	6.98E-05	1.523	1.218	1.905	2.28E-04
TUBB3	3.40E-04	1.520	1.143	2.023	4.00E-03
CCR5	1.42E-03	1.337	1.109	1.613	2.39E-03
CD86	1.67E-03	1.548	1.155	2.073	3.41E-03
ZBED2	4.70E-03	1.383	1.146	1.669	7.37E-04
CD8A	2.79E-05	1.295	1.128	1.487	2.41E-04
CD8B	1.63E-04	1.312	1.138	1.512	1.79E-04
HLA-DOA	2.30E-03	1.389	1.104	1.747	5.02E-03
CCDC74A	5.49E-04	2.492	1.653	3.757	1.29E-05
PTPN22	3.05E-04	1.442	1.177	1.765	3.99E-04
SERPINB9	1.09E-04	0.560	0.447	0.701	4.25E-07
SERPINB8	7.83E-05	2.370	1.546	3.633	7.48E-05
HK3	3.52E-03	1.537	1.163	2.032	2.52E-03
BATF	5.45E-03	1.398	1.139	1.715	1.32E-03
TNFAIP8L2	6.21E-05	1.714	1.252	2.347	7.77E-04
CHGB	2.63E-03	0.724	0.606	0.864	3.60E-04
CD5L	4.40E-07	1.246	1.094	1.419	9.36E-04
LAPTM5	1.84E-03	1.616	1.211	2.157	1.12E-03
B2M	9.52E-05	1.732	1.314	2.283	9.52E-05
LILRB5	2.97E-03	1.560	1.157	2.103	3.55E-03
LILRB2	6.59E-04	1.526	1.172	1.988	1.71E-03
GRAP2	1.16E-05	1.630	1.296	2.050	2.95E-05
ABCA17P	1.20E-04	0.638	0.484	0.842	1.48E-03
C9orf110	4.21E-03	1.451	1.104	1.908	7.69E-03
BIN2	3.87E-05	1.508	1.185	1.917	8.20E-04

RHBDL2	2.78E-03	1.760	1.264	2.452	8.23E-04
CD300A	2.13E-04	1.591	1.211	2.091	8.62E-04
MDGA1	9.29E-03	1.590	1.211	2.088	8.35E-04
MYCN	1.14E-03	1.688	1.238	2.302	9.33E-04
CD300LF	1.33E-03	1.598	1.246	2.050	2.19E-04
NINJ2	3.05E-05	1.907	1.329	2.738	4.65E-04
ATP6V0D2	9.49E-04	1.294	1.105	1.516	1.41E-03
PRDM1	4.32E-04	1.758	1.283	2.409	4.45E-04
PPM1K	2.01E-07	2.353	1.536	3.604	8.36E-05
SUSD2	1.13E-05	1.612	1.240	2.097	3.67E-04
KCNJ4	3.71E-05	1.433	1.158	1.775	9.54E-04
AGPAT4	4.20E-04	1.807	1.314	2.484	2.70E-04
RCSD1	1.39E-04	1.680	1.187	2.377	3.40E-03
FLI1	3.36E-03	2.098	1.406	3.131	2.88E-04
GPR27	4.98E-05	0.670	0.530	0.846	7.75E-04
SOCS2	1.19E-06	1.809	1.352	2.419	6.54E-05
CCNO	7.58E-04	1.791	1.280	2.506	6.71E-04
IL1R2	1.98E-05	1.626	1.280	2.066	6.87E-05
RNF43	5.32E-07	0.546	0.420	0.709	5.75E-06
ITPR2	2.00E-03	1.612	1.244	2.088	3.02E-04
FFAR3	5.22E-06	1.460	1.197	1.781	1.86E-04
ZNF883	7.68E-06	0.606	0.478	0.769	3.61E-05
IFNG	6.57E-03	1.372	1.134	1.660	1.13E-03
MTUS1	2.61E-06	0.514	0.389	0.679	2.77E-06
GSTO2	1.89E-05	0.591	0.436	0.801	6.98E-04
ME1	1.47E-05	1.506	1.214	1.868	1.94E-04
ERBB3	5.16E-07	0.662	0.517	0.847	1.06E-03
SLIT1	2.83E-04	1.359	1.120	1.648	1.89E-03
ZNF560	1.74E-04	0.649	0.495	0.849	1.64E-03
CCL3	6.26E-04	1.588	1.227	2.056	4.43E-04
NFATC2	5.78E-03	1.505	1.129	2.007	5.33E-03
NFATC4	2.37E-03	1.796	1.289	2.501	5.34E-04
CCL4	1.67E-04	1.452	1.195	1.765	1.74E-04

GCET2	2.59E-03	1.464	1.136	1.887	3.26E-03
MGLL	2.14E-03	1.718	1.382	2.137	1.13E-06
TRPM2	2.12E-03	1.657	1.244	2.207	5.59E-04
TGFBI	3.38E-05	1.949	1.360	2.793	2.80E-04
LCK	2.11E-04	1.345	1.153	1.569	1.57E-04
ADAM23	7.55E-03	1.391	1.135	1.705	1.45E-03
LTB	2.83E-04	1.417	1.178	1.705	2.14E-04
ZNF541	3.14E-04	0.548	0.393	0.764	3.85E-04
SSX1	5.60E-04	1.267	1.109	1.447	4.94E-04
FASLG	2.48E-03	1.352	1.142	1.600	4.51E-04
CCDC64	1.82E-07	1.297	1.088	1.547	3.69E-03
FAM105A	1.97E-04	2.143	1.489	3.084	4.04E-05
WWTR1	9.30E-03	1.559	1.149	2.116	4.37E-03
CCL18	2.90E-03	1.271	1.094	1.477	1.71E-03
ZNF677	7.96E-04	0.489	0.343	0.698	8.15E-05
MMP12	1.06E-06	1.420	1.240	1.626	3.94E-07
PRG2	7.64E-03	1.489	1.124	1.973	5.47E-03
NEDD9	7.30E-07	0.476	0.338	0.670	2.10E-05
LTA	6.28E-03	1.541	1.198	1.983	7.65E-04
IL32	1.46E-04	1.548	1.255	1.909	4.33E-05
ZNF391	5.05E-06	0.552	0.424	0.718	9.69E-06
SAMSN1	7.70E-03	1.320	1.085	1.606	5.59E-03
MGC29506	9.80E-03	1.270	1.091	1.479	2.06E-03
EMR1	5.43E-05	1.506	1.172	1.934	1.36E-03
AIM2	4.17E-03	1.347	1.114	1.629	2.16E-03
RNASE6	6.34E-04	1.643	1.165	2.317	4.62E-03
SSX5	2.94E-07	1.294	1.148	1.458	2.42E-05
P2RY6	1.76E-03	1.561	1.185	2.055	1.52E-03
GEM	5.65E-06	1.682	1.315	2.151	3.44E-05
LOC100190938	9.22E-05	0.615	0.488	0.775	3.75E-05
UCHL1	1.03E-04	1.365	1.104	1.689	4.13E-03
CASP1	5.55E-03	1.512	1.160	1.970	2.24E-03
NCF4	1.11E-03	1.799	1.281	2.525	7.00E-04

PCOLCE2	1.89E-04	0.631	0.499	0.797	1.12E-04
CNTNAP1	4.63E-04	1.891	1.289	2.773	1.11E-03
PAPSS2	4.27E-05	2.846	1.762	4.596	1.89E-05
DYSF	1.21E-04	1.634	1.239	2.155	5.12E-04
GATA6	2.26E-05	1.770	1.339	2.338	5.93E-05
SH2D1A	5.47E-04	1.334	1.137	1.564	3.95E-04
C4A	5.52E-03	1.342	1.097	1.642	4.27E-03
ANPEP	2.59E-06	2.071	1.544	2.778	1.16E-06
LAMA1	2.76E-05	1.833	1.412	2.379	5.24E-06
SLCO5A1	5.25E-04	1.512	1.241	1.843	4.07E-05
APOL4	1.70E-03	1.414	1.151	1.738	9.92E-04
APOL3	1.90E-04	1.381	1.155	1.651	3.95E-04
ST8SIA4	3.00E-03	1.543	1.186	2.008	1.24E-03
CD74	4.25E-03	1.489	1.173	1.891	1.09E-03
CD70	8.18E-03	1.227	1.051	1.433	9.81E-03
CD247	3.66E-04	1.424	1.176	1.724	2.98E-04
LILRA5	6.93E-03	1.478	1.103	1.980	8.84E-03
SLC25A21	8.75E-03	0.671	0.510	0.883	4.44E-03
DOCK11	1.22E-03	1.650	1.204	2.262	1.84E-03
GFRA2	3.91E-04	1.352	1.087	1.683	6.87E-03
FAM113B	6.64E-03	1.480	1.152	1.902	2.16E-03
ISG20	3.91E-07	2.027	1.511	2.720	2.44E-06
CD52	5.17E-03	1.469	1.181	1.826	5.45E-04
CD53	2.65E-03	1.479	1.129	1.937	4.46E-03
RAB31	3.64E-05	2.394	1.590	3.607	2.94E-05
CD163L1	1.15E-03	1.604	1.260	2.042	1.24E-04
ARMC9	4.51E-06	2.517	1.673	3.787	9.49E-06
CD40LG	9.29E-03	1.515	1.166	1.968	1.85E-03
MYEOV	3.50E-05	1.403	1.197	1.645	2.93E-05
HOMER2	5.38E-03	0.615	0.458	0.825	1.17E-03
SEL1L3	3.56E-06	1.506	1.226	1.850	9.54E-05
GPR114	2.02E-03	1.605	1.197	2.150	1.54E-03
AZGP1	9.60E-06	0.633	0.513	0.780	1.79E-05

F2RL3	1.21E-04	2.425	1.608	3.657	2.36E-05
CCL5	4.91E-03	1.448	1.202	1.746	1.01E-04
CD38	6.73E-03	1.334	1.097	1.621	3.79E-03
IFI27	3.87E-04	1.289	1.115	1.491	6.03E-04
PSD2	6.05E-05	1.396	1.177	1.656	1.32E-04
WNK4	7.12E-06	0.794	0.681	0.927	3.47E-03
CD3D	8.99E-05	1.323	1.149	1.523	9.68E-05
CD3E	1.89E-03	1.341	1.144	1.572	2.90E-04
CD3G	1.56E-03	1.346	1.128	1.605	9.50E-04
CD209	1.48E-05	1.559	1.206	2.014	6.93E-04
HCP5	1.86E-03	1.427	1.195	1.705	8.84E-05
ADAMTS14	1.73E-04	1.624	1.278	2.064	7.40E-05
RASGRP1	2.31E-03	1.230	1.053	1.437	8.91E-03
LOC100270746	1.80E-03	0.480	0.330	0.697	1.17E-04
LOC400759	3.85E-03	1.261	1.079	1.473	3.51E-03
CALHM2	4.16E-07	3.264	1.830	5.823	6.16E-05
FAM26F	3.76E-03	1.307	1.093	1.563	3.34E-03
HAVCR2	7.70E-04	1.484	1.108	1.987	8.10E-03
FXYD2	3.08E-03	1.381	1.134	1.682	1.31E-03
KREMEN2	7.26E-04	0.662	0.510	0.858	1.84E-03
CYTIP	2.16E-04	1.525	1.227	1.895	1.40E-04
FHDC1	4.13E-03	1.423	1.102	1.839	6.90E-03
TBC1D10C	2.07E-05	1.437	1.187	1.739	2.03E-04
TM7SF4	6.20E-04	1.298	1.129	1.493	2.57E-04
GADD45G	5.20E-05	1.396	1.142	1.707	1.13E-03
RIMS2	5.85E-05	1.242	1.116	1.381	7.13E-05
GRID1	2.41E-04	1.633	1.240	2.150	4.80E-04
BATF3	2.34E-05	2.507	1.666	3.772	1.04E-05
KLRK1	2.17E-03	1.348	1.133	1.603	7.50E-04
GNLY	3.27E-03	1.462	1.165	1.835	1.06E-03
IL15RA	2.62E-03	1.629	1.211	2.192	1.26E-03
SLC2A3	1.48E-03	1.529	1.168	2.001	1.98E-03
S1PR5	4.13E-03	1.930	1.286	2.896	1.50E-03

RAPGEF3	2.97E-06	0.550	0.407	0.743	9.84E-05
SCML4	7.98E-04	1.511	1.197	1.907	5.12E-04
ZNF208	5.44E-04	0.770	0.640	0.927	5.63E-03
GBP1	6.56E-04	1.259	1.106	1.434	5.07E-04
ST8SIA1	5.82E-04	1.474	1.202	1.808	1.94E-04
WAS	1.61E-03	1.597	1.181	2.160	2.39E-03
A4GALT	4.77E-04	1.867	1.244	2.803	2.60E-03
ZNF781	1.32E-04	0.745	0.598	0.928	8.76E-03
MPEG1	4.65E-04	1.686	1.202	2.367	2.51E-03
ADAMDEC1	3.75E-08	1.343	1.164	1.550	5.48E-05
CD7	4.95E-04	1.472	1.217	1.780	6.66E-05
SDC2	1.24E-03	1.529	1.215	1.924	2.98E-04
SLC8A1	4.94E-04	1.393	1.146	1.693	8.83E-04
C12orf75	9.23E-06	1.611	1.245	2.083	2.81E-04
AHNAK2	5.49E-05	1.714	1.259	2.332	6.13E-04
SCN4B	1.25E-05	1.659	1.269	2.169	2.13E-04
PLN	2.43E-04	1.260	1.115	1.424	2.09E-04
NCF1C	5.83E-03	1.463	1.148	1.864	2.11E-03
HTRA3	1.59E-06	1.845	1.385	2.459	2.90E-05
SPIB	2.15E-03	1.569	1.207	2.039	7.52E-04
RASGRP2	4.73E-04	1.784	1.318	2.414	1.76E-04
NLRC5	3.63E-03	1.526	1.216	1.914	2.65E-04
CXCR3	4.47E-03	1.351	1.142	1.598	4.55E-04
CXCR6	3.37E-03	1.330	1.126	1.570	7.80E-04
ZNF415	1.53E-05	0.696	0.583	0.831	5.93E-05
MYH7	8.52E-04	0.617	0.480	0.792	1.53E-04
THBS2	1.60E-03	1.597	1.282	1.990	2.95E-05
CELF5	1.63E-06	0.559	0.421	0.740	5.16E-05
CADM1	2.03E-06	1.469	1.209	1.786	1.10E-04
FGD2	1.08E-03	1.496	1.115	2.007	7.25E-03
CELF2	1.26E-05	1.766	1.318	2.367	1.41E-04
NKG7	5.13E-05	1.404	1.181	1.668	1.18E-04
CARD11	5.30E-08	1.741	1.354	2.239	1.57E-05

HCG26	3.56E-04	1.402	1.179	1.669	1.37E-04
ZNF667	2.36E-07	0.645	0.527	0.789	1.93E-05
AQP3	5.06E-04	1.733	1.298	2.314	1.92E-04
AQP1	1.91E-03	1.654	1.189	2.302	2.83E-03
EPHB2	3.77E-04	1.377	1.093	1.736	6.75E-03
C17orf60	8.22E-03	1.808	1.249	2.618	1.70E-03
PHYHIP	5.41E-03	1.362	1.097	1.692	5.09E-03
CLEC12A	2.87E-04	1.496	1.206	1.855	2.47E-04
TMEM150B	1.93E-05	1.627	1.260	2.101	1.93E-04
FEZ1	5.37E-05	1.476	1.168	1.865	1.11E-03
STEAP1	5.17E-03	0.607	0.448	0.821	1.19E-03
BCL11B	1.71E-04	1.500	1.229	1.829	6.36E-05
PALM3	4.32E-06	1.383	1.113	1.719	3.44E-03
UBASH3A	3.73E-03	1.359	1.132	1.631	9.76E-04
MAN1C1	1.07E-03	1.648	1.179	2.303	3.42E-03
SIT1	5.02E-04	1.365	1.155	1.614	2.59E-04
PARVG	1.52E-04	1.503	1.158	1.952	2.23E-03
PLEKHG4B	5.75E-06	2.117	1.527	2.935	6.83E-06
ZNF467	6.70E-07	1.584	1.210	2.073	8.20E-04
HPSE	1.98E-05	1.640	1.294	2.079	4.35E-05
SORL1	9.50E-04	1.516	1.216	1.891	2.18E-04
CD96	5.46E-06	1.384	1.157	1.656	3.73E-04
SKAP1	5.39E-03	1.386	1.137	1.689	1.24E-03
TRAT1	9.05E-05	1.388	1.159	1.662	3.57E-04
LINGO2	4.89E-05	0.621	0.486	0.793	1.33E-04
LINGO1	8.22E-03	2.132	1.403	3.242	3.96E-04
TNFRSF8	1.05E-05	1.525	1.214	1.916	2.85E-04
MALL	7.91E-03	1.409	1.117	1.777	3.76E-03
GBP2	8.89E-03	1.264	1.079	1.481	3.68E-03
ACSF2	2.32E-06	0.669	0.533	0.839	4.93E-04
GBP5	1.23E-03	1.257	1.094	1.443	1.21E-03
GBP4	8.72E-03	1.355	1.123	1.635	1.53E-03
CD6	3.23E-04	1.790	1.371	2.338	1.92E-05

CD2	4.72E-05	1.320	1.141	1.526	1.84E-04
VCAN	5.77E-03	1.571	1.160	2.127	3.48E-03
PEG3	6.14E-04	0.738	0.629	0.865	1.81E-04
ACY3	1.70E-03	1.421	1.096	1.843	8.01E-03
SIGLEC10	1.74E-03	1.388	1.127	1.709	2.01E-03
ARC	2.00E-03	1.649	1.321	2.059	1.01E-05
GLI2	4.95E-05	1.872	1.349	2.598	1.76E-04
MAFB	3.34E-04	2.113	1.408	3.173	3.08E-04
SLCO3A1	1.72E-05	2.166	1.484	3.161	6.18E-05
SLC2A6	7.20E-04	1.723	1.209	2.456	2.63E-03
CPS1	6.08E-04	0.748	0.630	0.888	9.40E-04
C9orf109	1.55E-03	1.611	1.194	2.175	1.81E-03
PLAU	9.47E-03	1.739	1.145	2.640	9.45E-03
ETV7	1.35E-03	1.313	1.104	1.561	2.10E-03
IKZF1	3.32E-04	1.666	1.276	2.174	1.75E-04
FOXD2	5.66E-05	1.698	1.237	2.331	1.05E-03
PLEKHG4	8.93E-07	0.436	0.302	0.631	1.05E-05
