# Supplementary material for: Identification of prognostic genes in uveal melanoma microenvironment
Source: PLoS One. 2020 Nov 16;15(11):e0242263. doi: 10.1371/journal.pone.0242263 (PMC7668584; doi:10.1371/journal.pone.0242263)
Supplement: S4 Table — (PDF) [file pone.0242263.s004.pdf]

S4 Table. 52 genes were significantly predicting the prognosis of UM patients in the validation cohort by both Kaplan-Meier and univariate Cox regression analyses (p-value < 0.01).

Gene	Kaplan-Meier analysis (p-value)	Univariate Cox regression analysis				Chromosome (GRCh38/hg38)
		HR	HR.95L	HR.95H	P-value	
ACSF2	1.59E-04	0.557	0.385	0.806	1.88E-03	17
ADCY1	5.90E-05	2.227	1.402	3.539	7.02E-04	07
AHNAK2	2.83E-04	1.460	1.199	1.778	1.65E-04	14
ALDH1L1	2.10E-03	0.361	0.198	0.656	8.36E-04	03
AZGP1	2.62E-03	0.706	0.585	0.853	3.06E-04	07
BCAT1	2.85E-03	1.817	1.252	2.637	1.67E-03	12
C1S	1.38E-03	1.436	1.091	1.891	9.87E-03	12
CA12	6.73E-06	1.481	1.245	1.761	9.05E-06	15
CALHM2	1.12E-04	1.531	1.198	1.956	6.60E-04	10
CELF2	1.45E-04	1.446	1.106	1.891	7.09E-03	10
CHAC1	1.54E-05	1.483	1.237	1.779	2.17E-05	15
ECM1	1.83E-03	1.354	1.121	1.636	1.68E-03	01
EDNRB	1.76E-03	0.798	0.685	0.929	3.68E-03	13
GDF11	9.66E-05	2.921	1.708	4.996	9.06E-05	12
GEM	4.80E-06	1.298	1.148	1.467	3.07E-05	08
GSTA3	1.58E-03	0.692	0.558	0.858	8.12E-04	06
HFE	4.19E-03	0.003	0.000	0.143	2.94E-03	06
HTR2B	3.42E-04	1.191	1.090	1.302	1.05E-04	02
HTRA1	7.17E-05	1.545	1.230	1.941	1.86E-04	10
IGFBP2	4.80E-05	1.518	1.246	1.850	3.51E-05	02
IL12RB2	7.02E-04	0.594	0.442	0.798	5.47E-04	01
ISM1	7.62E-04	1.934	1.408	2.655	4.59E-05	20
LINGO1	1.68E-03	2.016	1.242	3.271	4.53E-03	15
ME1	2.63E-03	1.543	1.115	2.136	8.86E-03	06
MTUS1	5.33E-04	0.538	0.397	0.729	6.33E-05	08
MYEOV	3.85E-03	1.316	1.124	1.540	6.49E-04	11
NEDD9	3.47E-03	0.306	0.165	0.570	1.91E-04	06
NR6A1	3.33E-03	0.043	0.004	0.454	8.91E-03	09
PAPSS2	5.99E-03	1.942	1.252	3.012	3.05E-03	10

PDE3A	7.79E-04	0.611	0.452	0.825	1.33E-03	12
PEG3	6.78E-03	0.745	0.598	0.927	8.25E-03	19
PLEKHG4B	1.19E-03	1.473	1.139	1.906	3.17E-03	05
PPM1K	5.97E-04	1.612	1.218	2.134	8.45E-04	04
RAB31	1.69E-03	1.505	1.154	1.963	2.55E-03	18
RAPGEF3	8.13E-04	0.413	0.223	0.764	4.86E-03	12
RIMS2	2.40E-03	1.440	1.213	1.708	3.01E-05	08
RNF43	7.98E-04	0.642	0.472	0.873	4.66E-03	17
SERPINB9	4.65E-03	0.654	0.495	0.864	2.80E-03	06
SIPA1L2	3.66E-03	1.924	1.341	2.762	3.84E-04	01
SKAP1	1.37E-03	0.000	0.000	0.000	8.57E-03	17
SLCO5A1	1.60E-03	1.412	1.124	1.772	2.99E-03	08
SMOC2	5.97E-03	3.321	1.790	6.160	1.40E-04	06
SOCS2	5.80E-03	3.020	1.485	6.141	2.28E-03	12
SSX1	6.40E-04	1.639	1.185	2.266	2.80E-03	X
SULF2	5.89E-04	1.924	1.321	2.802	6.48E-04	20
TGFBI	4.43E-03	1.256	1.056	1.494	9.83E-03	05
THBS2	2.07E-04	1.285	1.115	1.479	5.08E-04	06
TRPV2	1.86E-03	2.050	1.359	3.091	6.17E-04	17
WARS	2.07E-04	1.282	1.097	1.499	1.76E-03	14
ZNF415	2.22E-03	0.663	0.487	0.904	9.34E-03	19
ZNF667	8.28E-03	0.530	0.329	0.854	9.12E-03	19
ZNF781	2.27E-03	0.004	0.000	0.189	5.22E-03	19
